# Reprogramming of Tumor-reactive Tumor-infiltrating Lymphocytes to Human-induced Pluripotent Stem Cells

**DOI:** 10.1158/2767-9764.CRC-22-0265

**Published:** 2023-05-25

**Authors:** S.M. Rafiqul Islam, Takuya Maeda, Naritaka Tamaoki, Meghan L. Good, Rigel J. Kishton, Biman C. Paria, Zhiya Yu, Marta Bosch-Marce, Nicole M. Bedanova, Chengyu Liu, Michael J. Kruhlak, Nicholas P. Restifo, Raul Vizcardo

**Affiliations:** 1 Surgery Branch, NCI, NIH, Bethesda, Maryland.; 2 Center for Cell-Based Therapy, NCI, NIH, Bethesda, Maryland.; 3 Experimental Pathology Laboratory, NCI, NIH, Bethesda, Maryland.; 4 Transgenic Core, Division of Intramural Research, National Heart, Lung and Blood Institute, NIH, Bethesda, Maryland.; 5 CCR Microscopy Core Facility, NCI, NIH, Bethesda, Maryland.

## Abstract

Tumor-infiltrating lymphocytes (TIL) that can recognize and kill tumor cells have curative potential in subsets of patients treated with adoptive cell transfer (ACT). However, lack of TIL therapeutic efficacy in many patients may be due in large part to a paucity of tumor-reactive T cells in TIL and the exhausted and terminally differentiated status of those tumor-reactive T cells. We sought to reprogram exhausted TIL that possess T-cell receptors (TCR) specific for tumor antigens into induced pluripotent stem cells (iPSC) to rejuvenate them for more potent ACT. We first attempted to reprogram tumor neoantigen-specific TIL by αCD3 Ab prestimulation which resulted in failure of establishing tumor-reactive TIL-iPSCs, instead, T cell–derived iPSCs from bystander T cells were established. To selectively activate and enrich tumor-reactive T cells from the heterogenous TIL population, CD8^+^ PD-1^+^ 4-1BB^+^ TIL population were isolated after coculture with autologous tumor cells, followed by direct reprogramming into iPSCs. TCR sequencing analysis of the resulting iPSC clones revealed that reprogrammed TIL-iPSCs encoded TCRs that were identical to the pre-identified tumor-reactive TCRs found in minimally cultured TIL. Moreover, reprogrammed TIL-iPSCs contained rare tumor antigen-specific TCRs, which were not detectable by TCR sequencing of the starting cell population. Thus, reprogramming of PD-1^+^ 4-1BB^+^ TIL after coculture with autologous tumor cells selectively generates tumor antigen-specific TIL-iPSCs, and is a distinctive method to enrich and identify tumor antigen-specific TCRs of low frequency from TIL.

**Significance:**

Reprogramming of TIL into iPSC holds great promise for the future treatment of cancer due to their rejuvenated nature and the retention of tumor-specific TCRs. One limitation is the lack of selective and efficient methods for reprogramming tumor-specific T cells from polyclonal TIL. Here we addressed this limitation and present a method to efficiently reprogram TIL into iPSC colonies carrying diverse tumor antigen reactive TCR recombination.

## Introduction

Tumor-infiltrating lymphocytes (TIL) harvested from patient tumors can be expanded and infused into patients, resulting in the recognition and elimination of large established cancers including melanoma (1–4). TIL therapy has also been shown to mediate clinical responses in other solid tumor types including cervical cancer, gastrointestinal cancers, and breast cancer (5–8). T cells capable of recognizing patient-specific mutations, known as neoantigens, are thought to be responsible for the treatment effect of not only adoptive cell transfer (ACT; ref. 9) but also immune checkpoint blockers (10, 11). Various methods have been used to identify tumor-reactive or neoantigen-specific T-cell receptors (TCR) from TIL. CD8^+^ PD-1^+^ T cells in freshly isolated TIL are enriched for tumor-reactive/neoantigen-specific T cells (12, 13) as are CD39^+^ CD103^+^ T cells (14). Additional approaches include coculture of TIL with autologous tumor organoids (15) or autologous dendritic cells (DC) transfected with tandem minigenes (TMG) expressing patient-specific mutations or pulsed with mutant peptides to detect tumor neoantigen-specific T cells (7). Using these methods, neoantigen-reactive TCRs can be detected in most samples (16). However, in solid epithelial cancers other than melanoma, TIL products have not mediated clinical responses in most cases. This may be due in part to the high differentiation status of TIL, T-cell exhaustion, and an inability of expanded cells to persist after transfer. Previous findings suggest that less-differentiated T cells with enhanced stemness have superior antitumor efficacy (17). Patients who responded to TIL therapy received TIL products enriched with less-differentiated tumor-reactive T cells (18). Unfortunately, TIL in most human cancers is in an exhausted state and has limited potential to expand (12).

Given that the process of reprogramming exhausted T cells to induced pluripotent stem cell (iPSC) and redifferentiating them to T cells holds the promise of reversing T-cell exhaustion and differentiation, the utilization of this technology has been proposed as an alternative to overcome current ACT limitations. The fact that a single T cell forms a single clone of T cell–derived iPSC (T-iPSC) without altering the recombined structure of TCR genes, while gaining unlimited proliferation capability, makes this method conceptually ideal for cloning TCRs. However, it has been challenging to establish T-iPSCs with multiple tumor neoantigen-reactive TCRs directly from polyclonal TIL containing neoantigen-specific T cells along with numerous irrelevant bystander T cells (19, 20). Given that the generation of tumor antigen-specific iPSC colonies is technically difficult, most tumor-specific T-iPSCs have been established from highly clonal T-cell populations that were reactive to common tumor antigens (21, 22). Therefore, an additional step to selectively reprogram T-iPSC lines with desired TCRs from heterogeneous TIL is needed.

Here we present an optimal method to selectively reprogram tumor antigen-specific T cells from heterogeneous TIL populations by coculturing with autologous tumor cells and sorting the PD1^+^ 4-1BB^+^ CD8^+^ T-cell population before reprogramming. This distinctive method successfully identified tumor-reactive TCRs including low-frequency ones that were not detected with other conventional methods.

## Materials and Methods

### Study Approval

All experiments were conducted with the approval of the NIH Clinical Center and NCI Institutional Review Board and performed in accordance with NIH guidelines. All patients whose samples were studied in this article were enrolled into the Surgery Branch selected TIL protocol (03-C-0277) and if living, signed an informed consent form and received a patient information form before participation. All studies were conducted in accordance with The Declaration of Helsinki, The Belmont Report, and the U.S. Common Rule. All mouse experiments were approved under institutional animal study protocol by the Animal Care and Use Committee of the NCI.

### Lymphocyte Culture Media

Frozen TILs were thawed in complete media containing RPMI1640 medium (Lonza), 10% heat-inactivated human AB serum (catalog no. HP1022I, Valley Biomedical Inc.), 100 U/mL penicillin and 100 μg/mL streptomycin (Life Technologies), 2 mmol/L l-glutamine (Life Technologies), 10 μg/mL gentamicin (Quality Biological Inc.), 12.5 mmol/L HEPES (Life Technologies) with 6,000 IU recombinant human IL2 (Proleukin, Prometheus Laboratories Inc.) at 37°C in 5% CO_2_. All other healthy donor lymphocytes were cultured in the same media with 300 IU IL2.

### Isolation of Healthy Donor Peripheral Blood Mononuclear Cell and T-cell Subsets from Buffy Coat Bags

Buffy coat bags were obtained from the NIH blood bank prior to lymphocyte separation. Peripheral blood mononuclear cells (PBMC) were isolated using Ficoll-Paque PLUS (GE Healthcare Biosciences AB) according to the manufacturer's instructions. Harvested cells were stained with fluorescent-conjugated primary antibodies against CD3, CD4, CD8, CD62L, CD45RO, CD45RA, CCR7, and others listed in Table 1. The four T-cell subsets [Naïve, central memory (CM), effector memory (EM), and effector memory with CD45RA (EMRA)] were isolated on the basis of these markers; Naïve: CD62L^+^ CD45RO^−^ CD45RA^+^ CCR7^+^, CM: CD62L^+^ CD45RO^+^ CD45RA^−^ CCR7^+^, EM: CD62L^−^ CD45RO^+^ CD45RA^−^ CCR7^−^, EMRA: CD62L^−^ CD45RO^−^ CD45RA^+^ CCR7^−^. Sorted T-cell populations were collected, counted, and spun and were resuspended in CTL medium immediately after separation for reprogramming experiments or cryopreserved for further use for other experiments.

**TABLE 1:** The list of antibodies used in the study

Antibody	Catalog	Fluorochrome	Clone	Vendor
Anti-Human CD3	641397	APC-Cy7	SK7 (Leu-4)	BD Biosciences
Anti-Human CD8	335787	PE-Cy7	SK1	BD Biosciences
Anti-Mouse TCRβ	553171	FITC	H57-597	BD Biosciences
Anti-Human CD62L	555544	PE	DREG-56	BD Biosciences
Anti-Human CD45RO	559865	APC	UCHL1	BD Biosciences
Anti-Human CD4	566806	BV786	OKT4	BD Biosciences
Anti-Human CD45RA	563429	PerCp 5.5	HI100	BD Biosciences
Anti-Human CCR7	565869	BB515	3D12	BD Biosciences
Anti-Human CD25	563352	BV510	M-A251	BD Biosciences
Anti-Human CD56	563041	BV510	NCAM 16.2	BD Biosciences
Anti-Human TCRγδ	740179	BV510	B1	BD Biosciences
Anti-Human CD137 (4-1BB)	130-119-885	PE	4B4-1	Miltenyi Biotec
Anti-Human CD279 (PD-1)	17-9969-42	APC	MIH-4	e-Bioscience

### Subjects, TIL, and Autologous Tumor Lines

Frozen TIL samples were obtained from the Surgery Branch TIL lab repository. Patient information including age and sex is shown in Supplementary Fig. S1A. TILs were generated as described previously (7, 12, 13). Briefly, surgically resected tumors were cut into approximately 1–2 mm fragments and cultured in complete media containing high-dose IL2 (6,000 IU/mL). TIL fragment cultures from patients 1913 and 3784 were frozen after a short culture (days 13–16). TIL fragments from patient 4069 were further screened for neoantigen reactivity by TMG screening (7) and reactive TILs were expanded in the presence of irradiated feeder cells, 50 ng OKT-3 and 3,000 IU IL2 in 50-50 media (RPMI-AIM-V with 5% human AB serum with pen strep and l-glutamine) to reach approximately 100–150 billion cells for infusion and leftover cells were frozen. All patients had undergone prior therapies including surgery, chemotherapy, and immunotherapy.

Matched patients’ melanoma cell lines were established from enzymatically digested tumor specimens and kept in culture in RPMI1640 medium supplemented with 10% FBS (Gibco) at 37°C and in 5% CO_2_. Melanoma tumor cell lines were authenticated based on the identification of patient-specific somatic mutations and HLA molecules (12, 13). Cell lines confirmed negative for *Mycoplasma* tested frequently by using Cambrex MycoAlert *Mycoplasma* detection assay (Promega) according to manufacturer's instruction.

### *In Vitro* Activation of T Cells

Healthy donor PBMC or TILs were stimulated *in vitro* with plate-bound αCD3 Ab (100 ng/mL) or αCD3/28 dynabeads (Gibco) in a ratio of cells to beads 1:1 (according to manufacturer's instructions) in complete media (RPMI-1640+10% human serum) in the presence of 300 IU recombinant human IL2 for 4 days.

### TIL and Tumor Cell Coculture

TIL with minimal *in vitro* culture (13–16 days) were thawed in complete media in the absence of cytokines and rested overnight at 37°C and in 5% CO_2_. Tumor cell lines were washed with PBS once, trypsinized, spun down, and counted. TIL (1E+5 cells) were cultured with or without autologous tumor cell line (1E+5 cells) in 200 μL of tumor cell media (RPMI-1640+10% FBS) in a 96-well round-bottom plate (Corning, catalog no. CLS3367). Cells were cultured for 16 hours, harvested, stained with fluorochrome-labeled antibodies, and sorted for live CD3^+^ CD4^−^ CD8^+^ CD25^−^ CD56^−^ TCR gamma delta^−^ PD-1^+^ 4-1BB^+^ population by FACS Aria II (BD).

### Flow Cytometry and Cell Sorting

Fluorescently labeled antibodies used are shown below in Table 1. Cells were stained with propium iodide (PI) or fixable live/dead stain (Invitrogen) to exclude dead cells. Cells were incubated with antibodies for 30 minutes at 4°C and washed twice before sorting acquisition. The samples were analyzed by BD Fortessa and sorted by BD Aria II. Flow cytometry data were analyzed using FlowJo (RRID: SCR_008520). Data were gated on live cells (PI negative) and single cells. Gates were set based on fluorescence minus one control. The purity of sorted cells was usually more than 99%. The list of antibodies used is shown in Table 1.

### Reprogramming of Peripheral Blood T Cells to T-iPSCs

Human whole T cells from PBMC or sorted populations were subjected for reprogramming to T-iPSCs. T cells were stimulated as described above by αCD3 Ab or αCD3/28 dynabeads. Cells were transduced with CytoTune-iPS 2.0 Sendai Reprogramming Kits (Thermo fisher scientific) carrying Yamanaka factors (Oct4, Sox2, Klf4, and c-Myc) and SV40 (large T antigen) in a 24-well plate for 24 hours in complete media without cytokines. The next day, cells were washed with fresh media, spun down, replaced with T-cell media with IL2, and transferred onto laminin coated (iMatrix 511, reprocell catalog no. NP892-012) dishes for attachment. On the following day, media was changed to human embryonic stem cell media (StemFit) containing 50 ng/mL of bFGF (R&D) and continue to culture until embryonic stem (ES) cell-like colonies started to appear. Fresh media was added everyday with bFGF, and iPSC colonies were stained with alkaline phosphatase (AP) and counted typically at days 20–25.

### Reprogramming of TIL to TIL-iPSCs

TILs were stimulated as described above by αCD3 Ab, αCD3/28 dynabeads, or autologous tumor cells and sorted for the CD3^+^ CD4^−^ CD8^+^ PD-1^+^ 4-1BB^+^ population. Cells were transduced with CytoTune-iPS 2.0 Sendai Reprogramming Kits (Thermo Fisher Scientific) carrying Yamanaka factors (Oct4, Sox2, Klf4, and c-Myc) and SV40 (large T antigen) and cultured as described above. When ES cell-like colonies appeared around days 20–25, undifferentiated iPSC colonies were chosen manually using the microscope based on their morphology and individual iPSC colonies were transferred onto matrigel (BD) coated 6-well plates and allowed to expand in StemFit media with bFGF (50 ng/mL). Rock inhibitor (10 mmol/L Y-27632; Tocris catalog no. 1254) was used during passage and freeze down. At passage 3, individual TIL iPSC clones which survived and proliferated well were counted as established and frozen down until further characterization and experimental analysis.

### iPSC Colony Count and Measurement

Bright field images of colonies grown in 6-well plates were collected using a Zeiss AxioObserver Z1 microscope equipped with a 10x plan-apochromat (numerical aperture 0.45) objective lens, a condenser lens with 0.55 N.A., motorized stage and Hamamatsu ORCA Flash4 v2 sCMOS camera. Tile imaging mode in the Zen software was used to collect multiple images covering 80% of the good area, for each well in the 6-well plate. The tile images were stitched using the Zen software and the resultant image was analyzed using the image analysis module of the Zen software. The images were background subtracted, contrast inverted, and an intensity threshold applied to segment the cell colonies from the background. The area of the individual colonies was measured.

### iPSC Characterization

AP staining was performed using StemAb Alkaline Phosphatase Staining Kit II (catalog no. 00-0055) according to the manufacturer's instructions. Images were captured using phase contrast bright field microscopy (Zeiss).

For immunocytochemistry, cells were cultured on a glass bottom dish (Thermo Fisher Scientific) and fixed with 4% paraformaldehyde in PBS for 30 minutes. Cells were then washed with PBS, permeabilized, blocked, and incubated with antibodies (1:100) for 1 hour at room temperature per the manufacturer's protocol (Millipore SCR078). Following incubation, cells were washed with PBS and mounted with DAPI, and images were captured using a Zeiss LSM710 confocal microscope equipped with a 40x plan-apochromat (N.A. 1.3) objective lens (Carl Zeiss Microscopy, LLC).

For Spectral Karyotype (SKY) chromosome analysis, iPS cells were split a day before harvesting to enrich the number of cells in the growth phase. The next day, metaphase cells were arrested with colcemid (50 ng/mL; 4 hours; Life Technologies) were trypsinized, and underwent standard hypotonic treatment in 0.5% potassium chloride buffered with HEPES at 37°C for 20 minutes. Cells were fixed in fresh cold methanol-acetic acid (3:1). Metaphase spreads were prepared under optimized humidity conditions. SKY analysis was performed using Human Spectral Karyotyping Kit according to the manufacturer's protocol, using ASI Spectral cube and Hi-SKY software (Applied Spectral Imaging).

### Teratoma Formation

Human iPSC cells were dissociated with PBS (Thermo Fisher Scientific, catalog no. 10010023) supplied with 0.5 mmol/L Ethylendiaminetetraacetic acid (EDTA) (Thermo Fisher Scientific, catalog no. 15575020). Approximately 1E+7 cells were resuspended in 500 μL E8 (Thermo Fisher Scientific, catalog no.A1517001) medium supplied with 25 mmol/L HEPES (Thermo Fisher Scientific, catalog no.15630080). The cell suspension was cooled on ice, and then 50% volume (250 μL) of cold Matrigel (Corning, #354277) was added. The mixture was quickly injected subcutaneously into NSG mice (JAX, Stock #005557) at 150 μL per injection site (two sites per mouse). After 6–8 weeks, visible tumors were removed and fixed in 10% Neutral Buffered Formalin (Azer Scientific, catalog no. C871Y29). The fixed tumors were embedded in paraffin and stained with hematoxylin and eosin.

### TCRα and TCRβ Analysis

For bulk TIL, sorted T cells and iPSC (about 1E+5 cells per group) were spun down, washed with PBS once and snap frozen in 50–100 μL of 1 mol/L HEPES buffer (GIBCO#15630-080).

For TIL-iPSCs, cells were trypsinized, dissociated into single-cell suspension and counted in an automated cell counter machine (Countess). Approximately 1E+5 cells from 15–20 individual iPSC lines were mixed and pooled together into a 15 mL tube (master tube), spun down and washed once with PBS and snap frozen in 100 μL of 1 mol/L HEPES buffer. All the remaining colonies were trypsinized, collected, and frozen down (mother dish). Genomic DNA extraction and Immunoseq TCRβ survey sequencing were performed by Adaptive Biotechnologies. The results were analyzed using IMMUNOSEQ ANALYZER. For pooled samples the TCRs that are more than 0.5% of productive TCRs were considered to be valid. To identify the TCRα-TCRβ paired sequence, genomic DNA was extracted from individual TIL-iPSC clones and analyzed by Adaptive Biotechnologies.

### Construction of Candidate TCRs

To test the reactivity of candidate TCRs, retroviral vector constructs were synthesized and transduced into healthy donor T cells using the method described previously (7, 23). Briefly, TCRα V-J regions were linked to the mouse TCRα constant chain, and TCRβ-V-D-J regions were linked to the mouse TCRβ constant (CB2) chain. Mouse TCR constant regions promote pairing of the introduced TCR (24) and facilitates identification of positively transduced T cells by flow cytometry using an antibody specific for the mouse TCRβ constant chain (eBioscience, catalog no.: 47-5961-82 or BD Pharmingen, catalog no.: 553171). The mouse-constant regions were also modified to introduce additional disulfide bonds.

### Retroviral Transduction of TCR Gene

293 GP cells were plated on poly-D-lysine–coated plates at a concentration of 0.8 million cells per well in a 6-well plate with 2 mL of DMEM containing 10% FCS, glutamine, and HEPES buffer without antibiotics one day before transfections. Packaging cells were transfected with 1.5 μg of retroviral plasmid DNA encoding each TCR cloned into PMSGV1 along with 0.7 μg of helper plasmid RD114 using 10 μL Lipofectamine 2000 in OptiMEM (Invitrogen) for 8 hours. Medium was replaced 8 hours after transfection and cells were incubated for further 48 hours in complete media. To capture the viral particles, retroviral supernatants were spun at 2,000 × *g* for 2 hours at 32°C in 6-well non–tissue culture–treated plates coated with Retronectin (Takara Bio). Healthy donor peripheral blood lymphocytes were used as donor T cells for transduction. T cells were activated using complete media + 50 ng/mL OKT3 (Miltenyi Biotec) of media for 48 hours and 2 million cells were added per well of virus-coated 6-well plate, spun for 10 minutes at 300 × *g* at 32°C, then incubated overnight at 37°C. Surface murine TCRβ constant region^+^ cells were transduced with the cloned TCR pair successfully.

### Generation of Immature DCs as Antigen-presenting Cells

Monocyte-derived, immature DCs were generated on tissue culture tissue culture flasks. Briefly, apheresis samples were thawed, washed, counted approximately 5–10E+6 cells/mL with AIM-V media (Life Technologies) and then incubated at approximately 1E+6 cells/cm^2^ in an appropriately sized tissue culture flask and incubated at 37°C, 5% CO_2_. After 90 minutes, nonadherent cells were vigorously washed with AIM-V media and collected, and then incubated with AIM-V media for another 60 minutes. The flasks were then vigorously washed again with AIM-V media and then the adherent cells were incubated with DC media. DC media comprised of RPMI-1640 containing 5% human serum, 100 U/mL penicillin, and 100 μg/mL streptomycin, 2 mmol/L l-glutamine, 800 IU/mL GMCSF (Leukine), and 200 U/mL IL4 (Peprotech). On days 2–3, fresh DC media was added to the cultures with 200 U/mL of GMCSF and 200 U/mL IL4. DCs were used on days 5–6 after starting the culture.

### Peptide Pulsing

On day 5, DCs were harvested and then resuspended at 5E+5 cells/mL 200 U/mL of GMCSF and 200 U/mL IL4. Peptides were dissolved in DMSO and pulsed onto the antigen-presenting cells (APC) at 10 μg/mL and incubated for 4 hours at 37°C with 5% CO_2_. After 4 hours of peptide pulsing, APCs were washed twice with PBS prior to setup the coculture with T cells.

### Patient-derived Xenograft

Fresh tumor specimens from patients were chopped into small fragments of 2 mm in dimension. One fragment was implanted subcutaneously at the flank of an NSG mouse using a 20-gauge needle. Tumor growth was monitored weekly. Patient-derived xenografts (PDX) were harvested when their sizes were greater than 1 cm in dimension. Tumor cell lines derived from PDXs were cut into small fragments and placed in GentleMACS c-tubes containing 20 mL of RPMI+10% FBS. The tumor cells were then mechanically dissociated by GentleMACS dissociator (Miltenyi Biotec). The resulting cell suspension was run through a 100 μm cell strainer and washed once with culture media before being placed in tissue culture flasks. Media were refreshed every 2–3 days and cells were split when confluence reached up to 70%. The PDX-derived tumor cell lines were characterized and screened for HLA-I and DR expression by FACS (anti-pan HLA antibody W6/32 and anti-human DR antibody). For epithelial tumor cells, additional markers such as EpCAM and E-cadherin were also examined.

### Coculture of PDX-derived Tumor Cells with Neoantigen-specific TCR

One day prior to coculture, tumor cells were seeded into a 96-well plate in concentration of 1E+5 cells in 100 μL of culture media per well. Next day, 1E+5 T cells in 100 μL of RPMI-1640 +10% human AB serum per well were added to the tumor cells. The plate was incubated at 37°C, in 5% CO_2_ for 16 hours. Supernatants from the coculture wells were collected for IFNγ ELISA assay and nonadherent cells were harvested for surface 4-1BB expression by FACS.

### Target Cell Recognition Functional Assay

PB T cells of healthy donors transduced with GFP or TCR pairs identified in TIL-iPSCs were cocultured with autologous or allogeneic tumor cells in Effector: Target ratio of 1:1 for 16 hours. TIL which contains neoantigen-specific T cells were used as positive control. Nontransduced or GFP transduced cells were used as negative control. 4-1BB upregulation was used as a marker of target recognition by TIL and TCR transduced T cells. The frequencies of 4-1BB^+^ cells among mTCRβ^+^ cells were considered as the ratio of reactive cells. To be considered as reactive the 4-1BB upregulation had to be greater than 1%.

### IFNγ ELISA

Supernatants from the coculture experiments were harvested and evaluated for IFNγ secretion by ELISA according to manufacturer's instructions [Human Interferon Gamma Colorimetric ELISA kit (Invitrogen # EHIFNG)]. Briefly, sandwich ELISA analysis of human IFNγ was performed by loading 50 μL coculture soup in a precoated plate and incubated for 2 hours at room temperature. The plate were then washed at least five times and incubated with 50 μL per well of a mouse anti-human IFNγ biotinylated antibody (Invitrogen # M701B) at a concentration of 0.05 μg/mL for 2 hours at room temperature. The plate was washed and incubated with 100 μL per well of Streptavidin-HRP (Thermo Fisher Scientific # N504) at 1:7,000 dilution for 30 minutes at room temperature. Detection was performed using 1-Step Ultra TMB Substrate (Thermo Fisher Scientific # 34028) for 30 minutes at room temperature in the dark. The plate was then stopped with 0.16 mol/L sulfuric acid. Absorbances were read on a spectrophotometer at 450–550 nm.

### Reporter Cell Line Generation

Red fluorescent protein (RFP)-expressing tumor cell lines were generated by stably infected with NucLight Red lentivirus system (catalog no.: 4476, Essen Bioscience) for *in vitro* cytotoxicity measurement. Briefly, tumor cell lines TC-4069, TC-1913, and TC-3784 cells were transduced with Nuclight Red Lentivirus (EF1a, Puro) at multiplicity of infection of 5 with 8 μg/mL polybrene. After 24 hours at 37°C cells were washed with fresh media and cultured for an additional 7 days. Infected tumor cell lines were selected with 2 μg/mL puromycin in RPMI-1640 + 10% FBS.

### Determination of Proliferation and Apoptosis by Live Cell Imaging

Incucyte, live cell analysis system (Sartorius, Essen Bioscience), placed inside a conventional cell culture incubator at 37°C in 5% CO_2_, was used for real-time imaging of RFP-expressing tumor cells from each patient's tumors (TC-4069, TC-1913, and TC-3784). A total of 5,000 tumor cells were seeded in 100 μL of complete culture medium in the 96-well plate and rested overnight in a CO_2_ incubator to settle down. Next day, mTCRβ^+^ sorted TCR transduced (healthy donor PBL) T cells were counted, washed once with PBS and resuspended in complete medium and added at 100 μL per well on top of tumor cells at the effector-to-target ratio of 2:1 ratio. The plates were then placed into the Incucyte real-time cell imaging device and the red cell count per image was measured over time. Throughout the assay, both phase and fluorescent images were collected using phase contrast and red fluorescence channels with a 10 × object. Images were taken every 3 hours for 48–72 hours, and each condition was run in quadruplicate. Images were analyzed using IncuCyte 2021B software and data were generated using the GraphPad Prism statistical software (GraphPad Software). The data were reported as mean ± SD and each experiment was performed at least twice from two different healthy donor PBL transduced with appropriate TCRs.

### Statistical Analysis

All data were analyzed and presented as mean ± SD. Statistical analysis was performed using GraphPad Prism program 8.1.1 (RRID:SCR_002798 GraphPad Software Inc.). The replication information is described in each figure.

### Data Availability

Genomic and TCR sequencing were performed by Adaptive Biotechnologies. Derived data from these analyses are available from the corresponding author upon reasonable request. Raw data from the other assays presented in this study may also be requested from the corresponding author.

## Results

### TCR Stimulation is Necessary for Reprogramming of T Cells to Generate T-iPSCs

**FIGURE 1:**
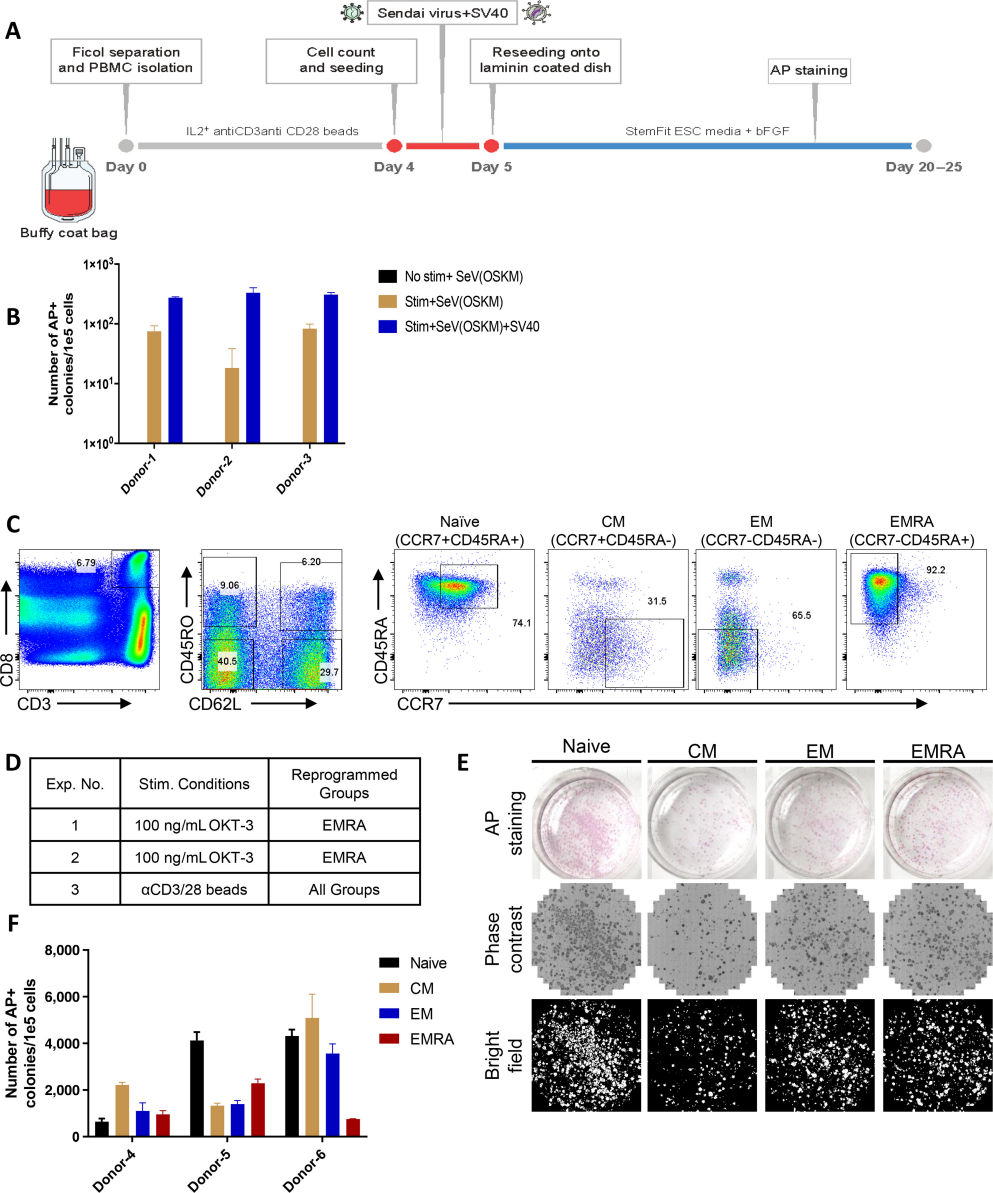
TCR stimulation is necessary for T cells to be reprogrammed to T-iPSCs. **A,** Schematic representation of PBMC derived T-cell reprogramming into iPSC. Whole PB T cells or the sorted populations were stimulated by αCD3/28 beads, and transduced with Sendai virus containing four Yamanaka factors and SV40 large T antigen. Approximately after 20–25 days’ culture in iPSC culture condition, the number of established iPSC colonies were counted. **B,** A bar graph indicates the numbers of AP-positive ES cell-like iPSC colonies derived from 1E+5 T cells with or without TCR stimulation. *N* = 3 for each donor. SeV containing four Yamanaka factors (OSKM) or OSKM and SV40 were used. **C,** Gating strategy to sort four subsets of CD8^+^ T cells (Naïve, CM, EM, and EMRA). PBMC were stained by antibodies against CD3, CD8, CD62L, CD45RO, CCR7, and CD45RA. CD8^+^ T cells were first gated on the basis of CD45RO and CD62L expression, followed by the second gate based on CD45RA and CCR7 expression. Four subsets of CD8^+^ T cells (Naïve, CM, EM, and EMRA) were sorted according to the gates indicated in the flow cytometry panels. **D,** A summary table showing which subset (Naïve, CM, EM, and EMRA) were reprogrammed to iPSCs by different stimulation methods. Sorted CD8^+^ T-cell subsets (Naïve, CM, EM, and EMRA) were stimulated by different methods (OKT-3 or αCD3/28 beads) before SeV infection. **E,** Representative AP, phase contrast, and bright field images were shown to describe the methods of iPSC colonies enumeration from different subsets (Naïve, CM, EM, and EMRA) of CD8 T cells reprogrammed into iPSC in each experiments following transduction of Sendai virus containing four Yamanaka factors and SV40 large T antigen. Each well was seeded with 1E+5 T cells. **F,** A bar graph indicates the numbers of AP-positive ES cell-like colonies derived from 1E+5 sorted CD8^+^ naïve/CM/EM/EMRA T cells activated by αCD3/28 beads. *N* = 3 for each donor.

To identify the optimal method of selective reprogramming of T cells with desired neoantigen-specific TCRs, we first optimized methods for reprogramming PB T cells. Given that cell-cycle progression is necessary for somatic cells to be reprogrammed to iPSCs (25, 26) and TCR engagement induces T-cell proliferation, we examined the necessity of TCR stimulation for efficient reprogramming of T cells. PB T cells were subjected to reprogramming with or without TCR stimulation by anti CD3/28 Ab. The efficiency of reprogramming toward an iPSC state was determined by the number of colonies successfully stained by the pluripotent stem cell marker, AP (Fig. 1A; ref. 27). Interestingly, T-iPSCs were established only when the T cells were stimulated (Fig. 1B). Moreover, although it is possible to generate a very limited number of colonies using only the four Yamanaka factors (c-myc, KLF4, Sox2, and OCT3/4), the addition of SV40 large T antigen greatly enhanced the efficiency of reprogramming (Fig. 1B). Therefore, TCR stimulation is essential for T-cell reprogramming.

Considering that the majority of TIL are differentiated cells and CD8^+^ T cells also contain other T-cell subsets (e.g., Naïve, CM, EM, and EMRA), we sought to elucidate whether TCR stimulation is necessary for the reprogramming of any T-cell subset (Fig. 1C). While there was a tendency that less-differentiated T cells have higher probability of reprogramming, all the subsets were able to be reprogrammed to T-iPSCs by prestimulation by αCD3/28 beads while only EMRA was reprogrammed by plate-bound OKT-3 (αCD3 Ab) stimulation (Fig. 1D–F) suggesting that bulk TIL containing different subsets can be reprogrammed to TIL-iPSCs by optimal TCR stimulation by αCD3/28 beads.

### Antigen Nonspecific TCR Stimulation of TIL Results in Reprogramming of Non-tumor Antigen-specific T Cells

**FIGURE 2:**
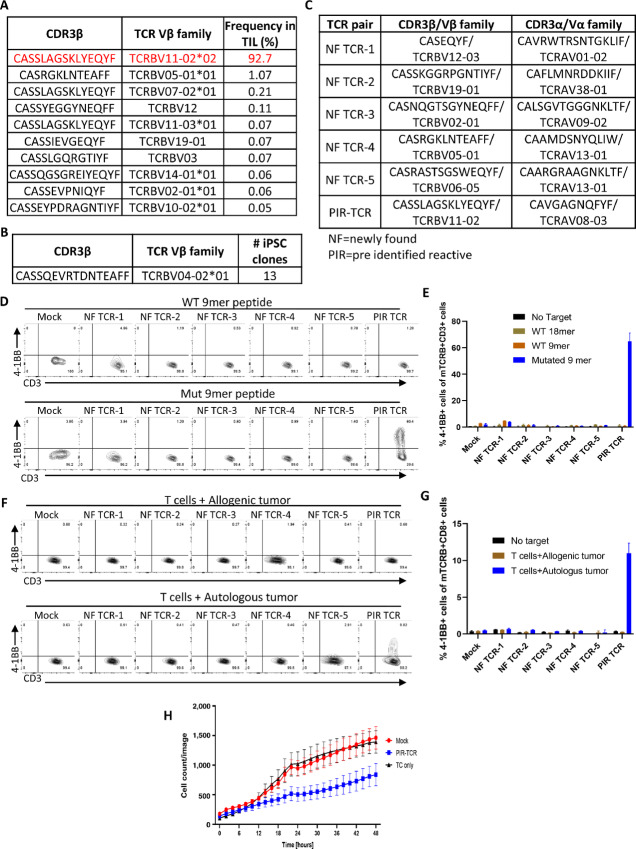
Nonspecific TCR stimulation by αCD3 Ab is not suitable for selective reprogramming of tumor-reactive T cells. **A,** A summary table of top 10 frequent TCRβ sequences in the starting cells, that is, expanded TIL of patient 4069, indicating the CDR3β amino acid sequence, Vβ family and frequency. The most frequent TCR was the patient's neoantigen-specific TCR (indicated in red). **B,** A summary table of TCRβ sequences of established TIL-iPSCs by αCD3 Ab stimulation, indicating the CDR3β amino acid sequence, Vβ family and the number of TIL-iPSC clones established. **C,** A summary table of six candidate TCR pairs including five newly found TCRs (NF TCR 1–5) and one PIR-TCR which were sequenced individually from master tube 6 containing 14 individual iPSC clones. The table indicates TCR pairs, CDR3β sequence, Vβ family, CDR3α sequence, and Vα family. NF, newly found TCR (unknown reactivity); PIR, preidentified reactive TCR. **D** and **E,** CD137 (4-1BB) upregulation assay of T cells transduced with candidate TCRα and TCRβ pairs identified from TIL-iPSCs (Fig. 2C). T cells were cocultured with autologous DC pulsed with wild-type (WT) peptide (16 mer or 9 mer) or mutant peptide (9 mer). D shows the representative FACS plots and E shows the percentage of 4-1BB^+^ cells in each condition. Representative data of three independent experiments from 3 different healthy donor T cells. Mock indicates empty vector transduced T cells used as negative control. **F** and **G,** CD137 (4-1BB) upregulation assay of T cells transduced with candidate TCRα and TCRβ pairs identified from TIL-iPSCs (Fig. 2C). T cells were cocultured with autologous, or HLA-matched allogeneic tumor cells derived from the PDXs model. F shows the representative FACS plots and G shows the percentage of 4-1BB^+^ cells in each condition. Representative data of three independent experiments from 3 different healthy donor T cells. Mock indicates empty vector transduced T cells used as negative control. **H,** A graph showing the real-time cell growth monitoring (cell count/image) of RFP-overexpressed PDX-derived tumor cell line cultured with T cells transduced with PIR-TCR analyzed by the Incucyte live imaging and analysis system (effector-to-target ratio of 2:1). Empty vector transduced T cells (Mock) and tumor cell alone (TC) were used as negative controls. The results are shown as mean ± SD (*N* = 4).

Given that T-iPSC can be generated from any T-cell subgroup we next tested whether this method can be applied to reprogramming tumor neoantigen-specific T cells identified in a patient with pancreatic cancer (Pt. 4069). To increase the probability of generating tumor neoantigen-specific TIL-iPSC clones, an almost monoclonal T-cell population (frequency 92.7%) expressing the TCR (TCRBV11-02*02) specific for the patient's neoantigen ZFYVE27 R6H (Fig. 2A) were activated by αCD3 Ab and reprogrammed as reported previously (21). A total of 13 different TIL-iPSC clones were established and TCRβ genes were sequenced. To our surprise, no clone carried the TCR specific for ZFYVE27 R6H. Instead, all the TIL-iPSC clones were derived from a single T-cell clone (TCRBV04-02*01) which was a very minor population and not detected by TCR deep sequencing in the original TIL products (Fig. 2B).

To establish neoantigen-specific TIL-iPSCs another TCR stimulation method, αCD3/28 beads stimulation was used before reprogramming. To acquire as many TCRs as possible from TIL-iPSCs, a total of 96 clones were isolated (Supplementary Fig. S1A). About 10–15 iPSC clones were pooled and mixed into one master tube (seven in total), total genomic DNA was extracted from each master tube, and TCRs were identified by TCRβ survey sequencing (Supplementary Fig. S1B). This modification of the stimulation method allowed TIL-iPSCs with preidentified neoantigen-specific TCR (TCRBV11-02*02) to be established along with 26 other TCRs (Supplementary Fig. S2A). We investigated whether the newly found TCRs (NF TCR) are specific to the patient's tumor or neoantigens. For that purpose, we chose one of the master tubes (master tube 6) having 14 different iPSC clones and their TCR α and β sequence was examined individually. We found five new TCR pairs along with the preidentified reactive one (PIR-TCR) in this tube (Fig. 2C; Supplementary Fig. S2A). We subsequently cloned these TCRs (Fig. 2C) and inserted them into a gammaretroviral vector and transduced into PB T cells of a healthy donor. The TCR transduced T cells were cocultured with the patient's autologous DCs pulsed with mutant or wild-type peptide (Supplementary Fig. S2B) and reactivity was analyzed by the expression of the activation markers 4-1BB and IFNγ secretion (Fig. 2D and E; Supplementary Fig. S2C). Though T cells with preidentified neoantigen-specific TCR (PIR-TCR) expressed 4-1BB and IFNγ, T cells with newly found TCRs (NF TCR 1–5) did not show any reactivity against wild-type or mutant peptides. Similarly, we have also investigated the specificity against autologous tumor cell lines derived from xenograft. All newly found TCR (NF TCR 1–5) pairs did not react upon coculture while PIR-TCR consistently recognized their target, expressed 4-1BB and secreted IFNγ (Fig. 2F and G; Supplementary Fig. S2D). Furthermore, we have also investigated whether this PIR-TCR can inhibit PDX-derived tumor cells proliferation by Incucyte live-cell analysis system (Sartorius, Essen Bioscience). Consistent with previous findings, PIR-TCR can suppress tumor growth (Fig. 2H).

Taken together, these data demonstrated that nonspecific stimulation by CD3 cross-linking is not always successful in specifically generating tumor-reactive TIL-iPSCs especially for those clones with low frequency, suggesting that a new method to selectively reprogram tumor-reactive T cells is needed.

### Tumor-reactive T Cells were Reprogrammed to iPSCs by Stimulating TIL with Autologous Tumor Cells and Enriching Reactive Populations with PD-1 and 4-1BB Expression

**FIGURE 3:**
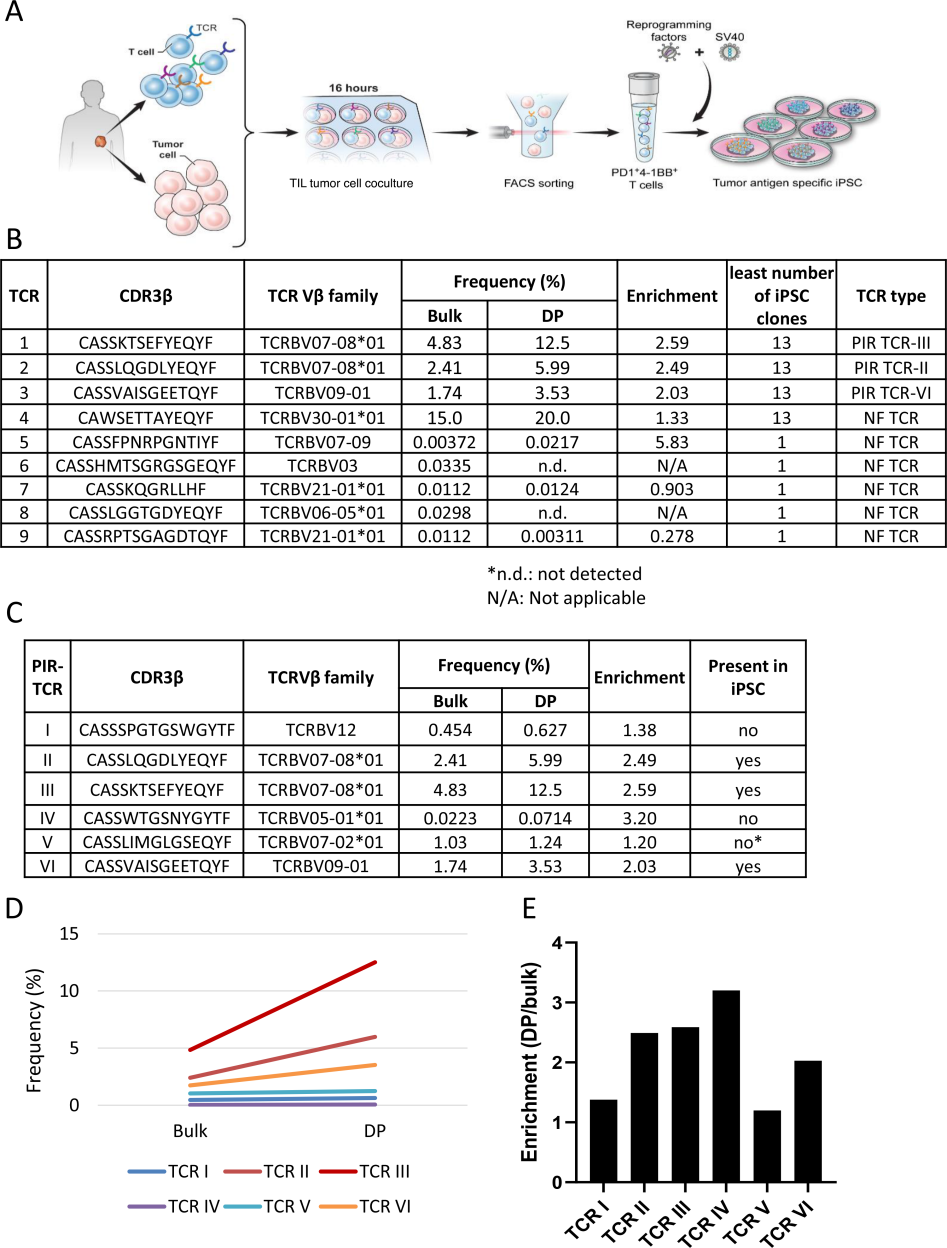
TIL-tumor cell coculture resulted in establishment of TIL-iPSCs from tumor-reactive T-cell clones. **A,** Schematic representation of TIL-tumor cell coculture to isolate tumor-reactive T cells and subsequent reprogramming into iPSCs. Autologous tumor cell line and minimally cultured TIL were cocultured for 16 hours, sorted for PD1^+^ 4-1BB^+^ CD8^+^ T cells, and transduced with Sendai virus containing four Yamanaka factors and SV40 large T antigen. On days 20–21 when cells formed domed shaped ES cell-like colonies, they were individually collected as clones and expanded. **B,** A summary table of nine different TCRs identified from 13 different master tubes containing 221 TIL-iPSC clones by using immunosequencing analysis. CDR3β amino acid sequence, Vβ family, frequency in bulk (before coculture in starting material) and in sorted PD1^+^ 4-1BB^+^ (DP) population, enrichment (DP/Bulk), the least number of iPSC clones established and the type of TCR were noted. PIR, preidentified reactive TCR; NF, newly found TCR (unknown reactivity). **C,** A summary table of the TCRβ sequence analysis of six preidentified tumor-reactive TCRs (PIR-TCR I–VI) indicating the CDR3β amino acid sequence, Vβ family, frequency in bulk (before coculture) and in the sorted PD1^+^ 4-1BB^+^ (DP) population after TIL-tumor cell coculture, enrichment (DP/Bulk) and presence in established iPSC clones. * means the TCR was not found in established T-iPSC clones but detected in the remaining clones in the dish after picking up iPS cell colonies (mother dish). **D,** A graph indicates the frequency of PIR-TCRs in starting cells (Bulk) and in sorted PD1^+^ 4-1BB^+^ (DP) cells. **E,** A bar graph indicates the enrichment analysis (the ratio of DP/Bulk) of those six PIR-TCRs.

Considering that T cells require TCR stimulation for reprogramming and tumor antigen-specific TILs are activated in the presence of autologous tumor cells, we sought to explore whether a coculture system could mediate selective reprogramming of tumor-reactive cells. To further enhance the probability of specific reprogramming of tumor-reactive TILs, we sorted on the basis of the activation markers PD-1 and 4-1BB following coculture (Fig. 3A; refs. 12, 23, 28, 29). For this study, we utilized melanoma TIL that had undergone minimal expansion *in vitro*, where autologous tumor cell lines were available and tumor-reactive TCRs were preidentified (13). TILs from patient 1913 were cocultured with their autologous tumor cell line for 16 hours and PD-1^+^ 4-1BB^+^ CD8^+^ T cells were sorted and infected with Sendai virus containing four Yamanaka factors and SV40 large T antigen to facilitate the reprogramming (Fig. 3A; Supplementary Fig. S3A). As a control, TILs stimulated with αCD3/28 beads were reprogrammed as well. After 3 weeks, typical ES cell-like colonies appeared and 221 colonies were manually collected from the dish prestimulated with autologous tumor cells for iPSC line establishment (Supplementary Fig. S1A). About 20 iPSC clones were pooled and mixed per master tube (13 in total), total genomic DNA was extracted from each master tube, and TCRs were identified by TCR β sequencing (Supplementary Fig. S1B). For control TIL-iPSCs generated by αCD3/28 beads stimulation, all the colonies were collected without cloning each individual colony and TCRs were identified by TCR sequencing. A total of nine different TCRs were detected from these 13 master tubes (Fig. 3B; Supplementary Fig. S3B). Four of them were present in all samples and the remaining five TCRs were detected only in one of the master tubes (Fig. 3B). Six tumor-specific TCRs had previously been identified from this patient and all six were detectable in the starting TIL and sorted PD-1^+^ 4-1BB^+^ population in various frequencies (Fig. 3C), Therefore, tumor antigen specific T cells can be successfully reprogrammed by autologous tumor cell coculture. The frequency of the six preidentified (PIR) TCRs were enriched in the PD-1^+^ 4-1BB^+^ population compared with the starting bulk population, showing that the enrichment of PD-1^+^ 4-1BB^+^ populations before reprogramming is a feasible strategy to pre-enrich antigen-specific clones (Fig. 3D and E). Three out of six PIR-TCR were present in relatively high frequency (>2%) in the PD-1^+^ 4-1BB^+^ population and these TCRs were detected in all master tubes suggesting a high number (at least 13) of iPSC clones carrying each PIR-TCRs (Fig. 3B). Another three PIR-TCRs were detected in PD-1^+^ 4-1BB^+^ population in relatively low frequency (<2%) and were not detected in any of the master tubes. One of the TCR which was not present in established TIL-iPSCs (TCR-V) was detected in the remaining dish after picking up iPSC colonies, suggesting the selection of more colonies may have resulted in detection.

**FIGURE 4:**
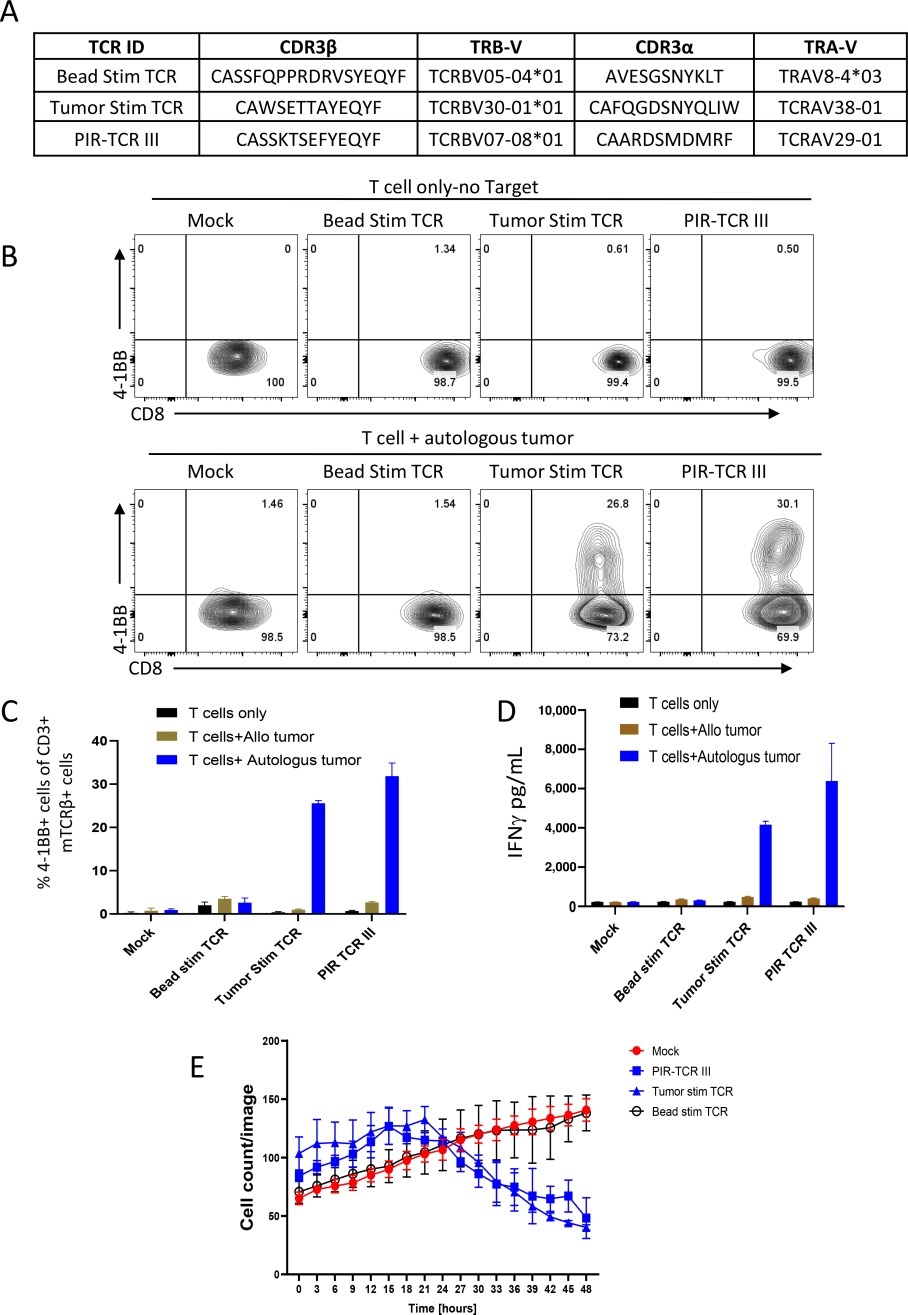
TIL stimulated with autologous tumor cell line generated antigen specific iPSC whereas αCD3/28 beads mediated stimulation did not. **A,** Summary of three candidate TCR pairs identified in TIL-iPSC lines established by different stimulation methods. Bead Stim TCR: The most dominant clone in the TIL-iPSCs established by αCD3/28 beads stimulation, Tumor Stim TCR: The TCR identified in the TIL-iPSC clones established by coculture with autologous tumor cells (TCR 4 in Fig. 3B), and PIR-TCR III: preidentified reactive TCR clone detected in the TIL-iPSC clones established by coculture with autologous tumor cells. The table includes TCR type, CDR3β amino acid sequences, Vβ family, CDR3α amino acid sequences, Vα family are described. **B** and **C,** CD137 (4-1BB) upregulation assay of T cells transduced with candidate TCRα and TCRβ pairs identified from TIL-iPSCs (Fig. 4A). T cells were cocultured with autologous or allogeneic tumor cell lines. B shows the representative FACS plots and C shows the percentage of 4-1BB^+^ cells in each condition. *N* = 3 for each condition. Representative data of four independent experiments. Mock: empty vector transduced PBL as a negative control, PIR-TCR III as a positive control. **D,** A bar graph showing the results of ELISA IFNγ production assay of T cells transduced with candidate TCRα and TCRβ pairs identified from TIL-iPSCs (Fig. 4A). T cells were cocultured with autologous or allogeneic tumor cell lines for 16 hours and culture soup were analyzed for IFNγ by ELISA. *N* = 3 for each condition. Representative data of four independent experiments. **E,** A graph showing the real-time cell growth monitoring (cell count/image) of RFP-overexpressed tumor cells from pt. 1913 cocultured with different TCRs (listed in Fig. 4A), analyzed by the Incucyte live imaging and analysis system at effector-to-target ratio of 2:1. Mock indicates empty vector transduced T cells used as a negative control. The results are shown as the mean ± SD of *N* = 4 each condition. A representative data of four independent experiments.

TIL-iPSCs established by αCD3/28 beads stimulation were almost clonal (Supplementary Fig. S3C) but this TCR did not appear in the list of tumor-reactive clones by previous studies (Fig. 3C; ref. 12). We investigated whether this TCR is reactive to autologous tumor cells. We also investigated whether the newly found TCRs in T-iPSCs established by TIL-tumor cell coculture, especially TCR-4 of Fig. 3B, which was detected in all 13 master tubes, is reactive or not. For that purpose, TCRα and TCRβ sequences of those T-iPSC clones were identified (Fig. 4A) by immunosequencing. TCRα and TCRβ pairs were cloned into a gamma retrovirus vector (pMSGV1) and transduced into healthy donor PB T cells and tested for specific recognition of autologous tumor cells. While T cells transduced with the TCR from beads stimulated T-iPSC did not express 4-1BB nor produced IFNγ, those with the TCR from tumor cell-stimulated T-iPSC expressed 4-1BB and produced IFNγ as PIR-TCR (Fig. 4B–D). Moreover, the T cells transduced with the TCR from TC-stimulated T-iPSC (Fig. 3B, TCR-4) showed cytotoxicity against autologous tumor cells similarly to PIR TCR-III, but those with the TCR from beads stimulated T-iPSC did not (Fig. 4E). These results demonstrated that TIL-tumor cell coculture before reprogramming is a better method to reprogram tumor antigen–specific TIL than nonspecific T-cell stimulation by αCD3/28 beads.

### T Cells with Tumor Antigen–specific TCRs of Extremely Low Frequency were Reprogrammed to TIL-iPSCs

**FIGURE 5:**
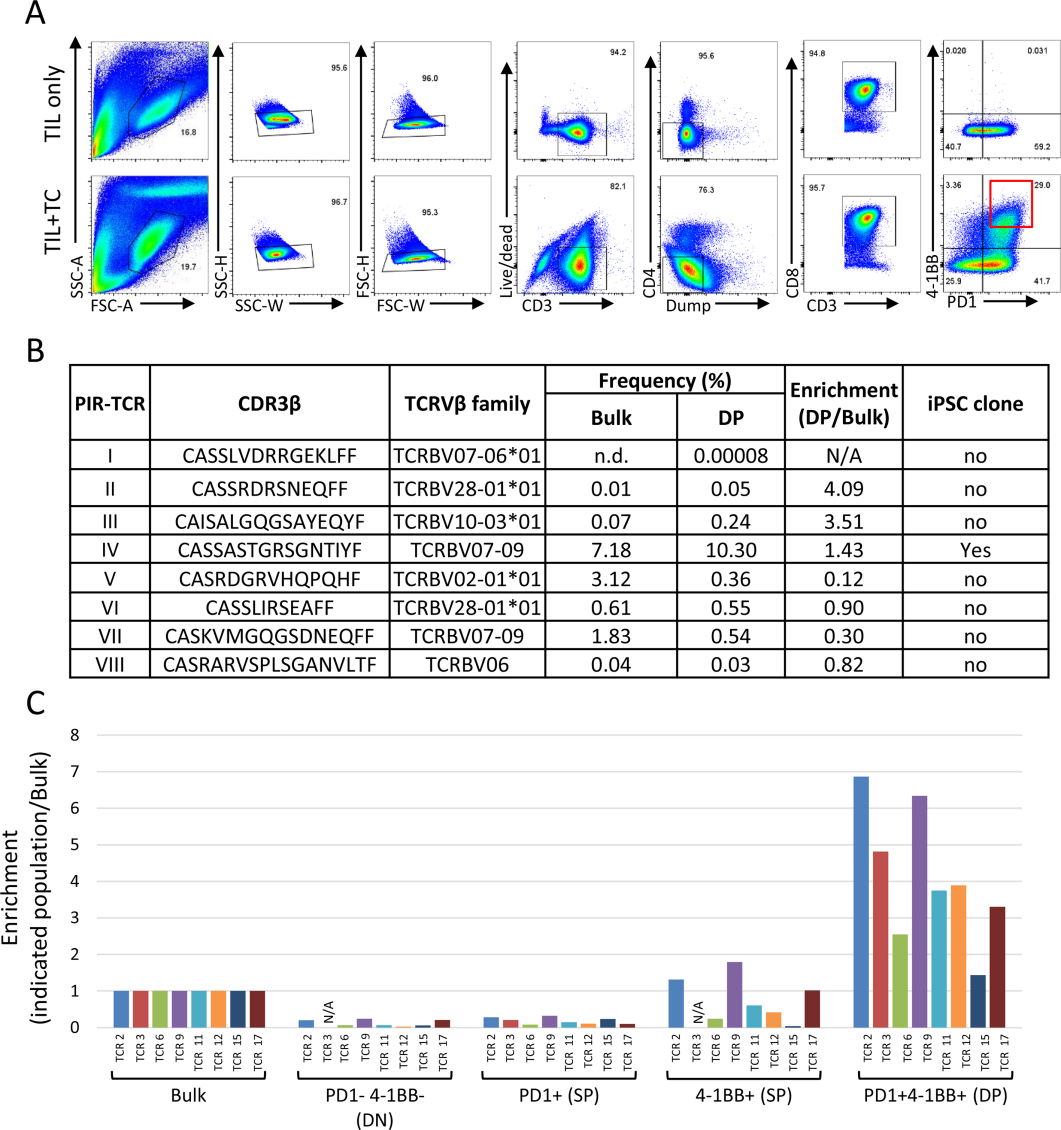
TIL-tumor cell coculture activated tumor-reactive T cells and generated tumor-reactive TIL-iPSCs from TIL of patient 3784. **A,** FACS panels showing the gating strategy to sort PD1^+^ 4-1BB^+^ CD8^+^ T cells after TIL-tumor cell coculture. Top panel shows the phenotype of TIL without coculture. Dump gate is a mixture of different T lineage markers (CD25, CD56, and TCRγδ) to exclude regulatory T cells (CD25^+^), natural killer cells (CD56^+^), and gamma-delta T cells (TCRγδ^+^). **B,** A summary table of the TCRβ sequence of eight preidentified tumor-reactive TCRs indicating their CDR3β amino acid sequence, Vβ family, frequency in bulk (before coculture) and in sorted PD1^+^ 4-1BB^+^ (DP) population, enrichment (DP/bulk) and presence in established iPSC clones. **C,** A bar graph showing the relative frequency of TCR clones, which were present in bulk population (starting cells) and reprogrammed to iPSCs, in four sorted populations [PD1^−^ 4-1BB^−^ (DN), PD1^+^ 4-1BB^−^ (PD1^+^ SP), PD1^−^ 4-1BB^+^ (4-1BB^+^ SP), PD1^+^ 4-1BB^+^ (DP)] after TIL-tumor cell coculture relative to bulk frequency of each TCR clone before coculture. Please see Supplementary Table S1 for the details of the TCR clones (TCR 2, 3, 6, 9, 11, 12, 15, and 17).

To confirm the TIL-tumor cell coculture can selectively reprogram tumor-reactive TIL, another melanoma TIL from patient 3784 was similarly cocultured with an autologous tumor cell line for 16 hours. PD-1^+^ 4-1BB^+^ CD8^+^ T cells were then sorted and reprogrammed into iPSCs (Fig. 5A). A total of 178 iPSC lines were established (Supplementary Fig. S1A) from this patient and TCRβ sequences were identified as described previously (Supplementary Fig. S1B). Of them, we found the preidentified tumor antigen–specific iPSC clone (clone 159) among the 178 colonies that were picked up and established. Furthermore, to demonstrate a successful reprogramming, we have examined clone 159 pluripotency by immunofluorescent staining of key genes associated with pluripotency (Supplementary Fig. S4A), chromosomal karyotyping for genomic stability (Supplementary Fig. S4B) and their ability to differentiate into three embryonic germ layers (Supplementary Fig. S4C). Together, these data confirm that antigen specific TIL iPSC attain true pluripotency.

This patient is a complete responder for TIL therapy and eight tumor-reactive TCRs were preidentified in this patient's sample using various methods (12, 13). Although most of the PIR-TCR were found at relatively low frequency in the starting bulk TIL and PD-1^+^ 4-1BB^+^ populations (Fig. 5B), one major TCR, which was enriched from 7.18% to 10.3% (1.43 times) in the PD-1^+^ 4-1BB^+^ compared with bulk population, was detected in two iPSC master tubes out of nine tubes (Fig. 5B). Even though only one PIR-TCR was reprogrammed (Fig. 5B, PIR-TCR IV), we identified a total of 25 different TCRβ chains from nine master tubes (Supplementary Table S1). Interestingly, most of the TCRs identified in TIL-iPSC clones were undetectable or at very low frequency in the starting TIL or PD-1^+^ 4-1BB^+^ population. Some were highly enriched in the PD-1^+^ 4-1BB^+^ population compared with the bulk population (Fig. 5C).

**FIGURE 6:**
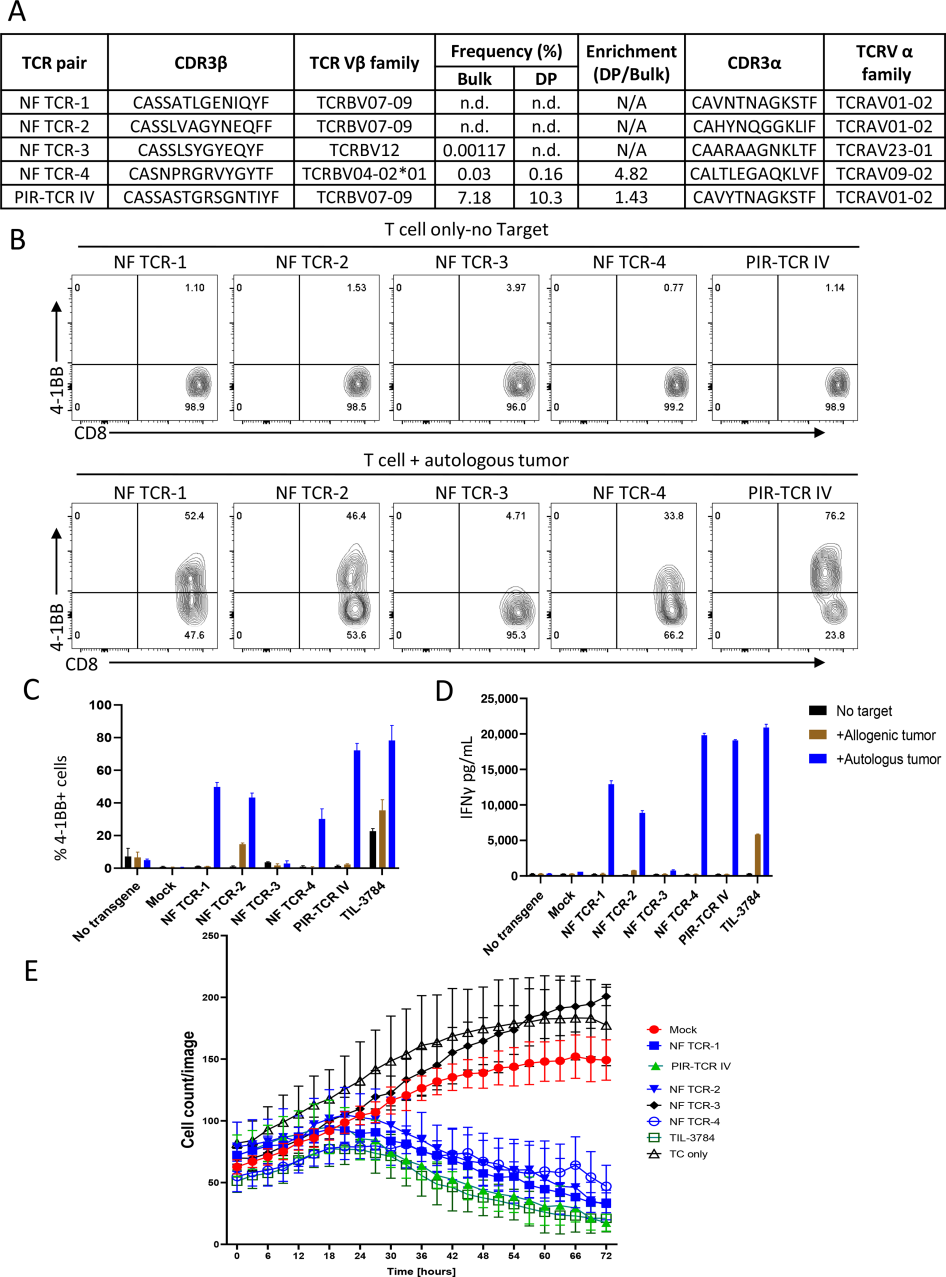
T cells with tumor-reactive TCRs of extremely low frequency were reprogrammed to TIL-iPSCs. **A,** Summary of five candidate TCR pairs including four newly found TCRs (NF TCR 1–4) and one PIR-TCR (IV) identified in TIL-iPSCs which were sequenced individually. The table includes CDR3 amino acid sequences of α and β chains, Vα family, Vβ family, and immunosequencing analysis of TCRβ which contains frequency in bulk (before coculture) and in sorted DP population after coculture, and enrichment (DP/Bulk). **B** and **C,** CD137 (4-1BB) upregulation assay of T cells transduced with candidate TCRα and TCRβ pairs identified from TIL-iPSCs (Fig. 6A). T cells were cocultured with autologous or allogeneic tumor cell lines. B shows the representative FACS plots and C shows the percentage of 4-1BB^+^ cells in each condition. Representative data of four independent experiments. Control PBL; no transgene, Mock indicates empty vector transduced T cells used as a negative control, TIL; expanded TIL containing tumor-reactive T cells. **D,** A bar graph showing the results of ELISA IFNγ production assay of T cells transduced with candidate TCRα and TCRβ pairs identified from TIL-iPSCs (A). T cells were cocultured with autologous or allogeneic tumor cell lines for 16 hours and culture soup were analyzed for IFNγ by ELISA (*N* = 3). Representative data of four independent experiments. **E,** A graph showing the real-time cell growth monitoring (cell count/image) of RFP-overexpressed tumor cells from pt-3784 cocultured with different TCRs (listed in A), analyzed by the Incucyte live imaging and analysis system in an effector-to-target ratio of 2:1. Mock indicates empty vector transduced T cells used as a negative control; TC only indicates tumor cells alone, and four newly found candidate TCRs (NF TCR 1–4) and a PIR-TCR IV were used for coculture and their antitumor properties were measured over 72 hours time period. The results are shown as mean ± SD of *N* = 4 each condition. A representative data of four independent experiments.

Because our previous experiments demonstrate the ability to generate several different tumor antigen–specific TCRs from a bulk TIL population by a coculture system before reprogramming, we sought to determine whether the unknown minor T-cell clones were preferentially reprogrammed to TIL-iPSCs because they were tumor antigen specific. To test this hypothesis, we sequenced TCRα and TCRβ chains from several TIL-iPSC lines and identified the TCRα and TCRβ chains (Fig. 6A). Three TCR pairs from T-iPSC clones whose TCRβ were not detected in the PD-1^+^ 4-1BB^+^ population, one TCR pair from a clone whose TCRβ was very low frequency (0.16%; Supplementary Table S1), and the PIR-TCR IV (Fig. 5B) as positive control were tested for specificity against autologous tumor cells. TCRα and TCRβ pairs were cloned into a gamma retrovirus vector (pMSGV1) and transduced into healthy donor PB T cells and tested for specific recognition of autologous tumor cells. Three out of four newly tested TCR pairs (NF TCR-1, 2, and 4) showed reactivity against autologous tumor cells as PIR-TCR IV (our positive control) by 4-1BB expression, cytokine production and target cell killing (Fig. 6B–E). These results demonstrate that TIL-tumor cell coculture before reprogramming can selectively enrich antigen-specific T cells and subsequently reprogram tumor-reactive T cells even from very minor clones.

## Discussion

Although iPSC technology is a new avenue to generate an unlimited amount of undifferentiated and nonexhausted populations of *de novo* T cells, the development of a method capable of reprogramming polyclonal tumor antigen-specific TIL is still necessary (21, 22, 30).

Several reports have already been published demonstrating the importance of enriching tumor and neoantigen-specific T cells for successful cell therapies against cancers (9, 31). However, it is always challenging to start with a polyclonal (bulk) population of T cells where mutation or antigen-specific TCR is unknown. Therefore, we started with some extensively studied patients where the mutation and their appropriate TCRs are known. Here we describe the first report of selectively establishing multiple tumor antigen specific TIL-iPSCs from patient TIL with minimal *in vitro* culture. The method to preferentially reprogram tumor-reactive T cells from heterogeneous populations of TIL is essential for clinical application of this strategy. We determined that while TCR stimulation is necessary for T-cell reprogramming, nonspecific TCR stimulation with αCD3 Ab does not always selectively generate tumor-reactive TIL-iPSCs even from highly enriched populations of tumor-reactive TILs. Consequently, we employed a tumor mediated stimulation to selectively reprogram tumor-reactive T-cell clones. To further enhance specific reprogramming of tumor-reactive TIL, we enriched PD-1^+^ 4-1BB^+^ cells after tumor cell coculture. This population was chosen due to previous demonstration that PD-1^+^ TIL contains neoantigen-specific TCRs (12, 13) and that tumor neoantigen-specific TCRs were identified from 4-1BB^+^ cells after TIL-TMG–expressing DC coculture (23). Most of the preidentified tumor antigen–specific T-cell clones were modestly enriched in the PD1^+^ 4-1BB^+^ population compared with starting TIL (Fig. 3C–E and 5B), and some of them were reprogrammed into TIL-iPSCs. Moreover, many TIL-iPSCs of unknown specificity were established from very minor clones, some of which were not detectable in starting TIL or the sorted PD1^+^ 4-1BB^+^ population by TCR sequencing. We confirmed the majority of tested TCRs were reactive and have cytotoxicity against autologous tumor cells. These data show that not only does the frequency of the reactive T-cell clones play a role, but also some other factors of T cells such as differentiation status, degree of exhaustion, and metabolic fitness profoundly influence which clones may be reprogrammed. Although it is difficult to address this question, our experiments suggested that proper TCR stimulation is the key for successful selective reprogramming of tumor-reactive T cells. TIL-iPSCs may derive from a relatively minor population which are enriched in tumor-reactive clones.

Recently another group reported establishment of TIL-iPSCs from TILs sorted by the expression of CD107a or 4-1BB after coculturing with autologous tumor organoids (32). However, their method is to first establish reactive TIL lines by nonspecific stimulation by αCD3 Ab before reprogramming to TIL-iPSCs, which we report here as not optimal (Pt. 4069, Fig. 2). The key points for selective reprogramming of tumor-reactive T cells are transducing reprogramming factors to TILs directly after TIL-tumor cell coculture and avoiding nonspecific TCR stimulation.

The frequency in the starting TIL population has been the most important factor for successful cloning of tumor antigen–specific T cells and their TCR genes. Even using novel technology to identify TCRα and TCRβ chains from a single cell, the detection limit would be one in a thousand cells, due to the throughput capacity (33–36). Our method is distinctive in that it has the potential to detect extremely minor tumor-reactive T cells from bulk TIL. Those TILs are specifically activated and reprogrammed into iPSC when they come in contact with their physiologic neoantigens. However, nonspecific activation by αCD3 Ab or bystander stimulation by other stimuli such as viral antigens does not selectively generate antigen-specific T cells.

The caveat of this method is the availability of autologous tumor cells. As an alternative, it may be possible to use novel technology like tumor organoids or autologous DC transduced with TMGs which contain the patient's tumor specific mutations as minigenes. Another caveat of not only our strategy but the whole field of differentiating T cells from iPSCs is the lack of a robust method to generate less differentiated/naïve-like T cells from human iPSCs, which is an area of research under focus by a number of groups (22, 37–39). Given that the potency of the differentiated T cells from human iPSCs still be controversial, in this study we focused on the method to selectively reprogram tumor-reactive T cells in TIL to TIL-iPSCs. To further demonstrate TCR antitumor specificity and function, we use healthy donor's PB T cells to transduce and evaluate the cloned TCR from TIL-iPSCs. If TIL-iPSCs with tumor-reactive TCRs established by this method are induced to generate less-differentiated naïve-like T cells, these rejuvenated T cells will have polyclonal tumor-reactive TCR population, high expansion capacity, and potential to persist longer *in vivo*, which may revolutionize the current adoptive cell–based therapies against cancers (40) and other diseases. Alternatively, tumor reactive TCRs found by this method can be directly cloned into virus vectors and transduced to PB T cells for immediate clinical application.

## Supplementary Material

Table S1 Table S1 indicating TIL-iPSCs were established from T cells of low frequency along with major tumor neoantigen specific T cells from patient 3784

Figure S1 Figure S1: A summary table of the patient samples used and the schema of the study

Figure S2 Figure S2: Newly identified TCRs in TIL-iPSCs established from patient 4069 TIL by αCD3/28 Ab stimulation

Figure S3 Figure S3: TIL-Tumor cell co-culture resulted in establishment of TIL-iPSCs from tumor reactive T cell clones from patient 1913

Figure S4 Figure S4: Characterization of tumor antigen specific TIL-iPSC clone derived from patient 3784

## References

[1] Rosenberg SA , Dudley ME. Adoptive cell therapy for the treatment of patients with metastatic melanoma. Curr Opin Immunol 2009;21:233–40. 19304471 10.1016/j.coi.2009.03.002 PMC3459355

[2] Besser MJ , Shapira-Frommer R, Itzhaki O, Treves AJ, Zippel DB, Levy D, . Adoptive transfer of tumor-infiltrating lymphocytes in patients with metastatic melanoma: intent-to-treat analysis and efficacy after failure to prior immunotherapies. Clin Cancer Res 2013;19:4792–800. 23690483 10.1158/1078-0432.CCR-13-0380

[3] Andersen R , Donia M, Ellebaek E, Borch TH, Kongsted P, Iversen TZ, . Long-lasting complete responses in patients with metastatic melanoma after adoptive cell therapy with tumor-infiltrating lymphocytes and an attenuated IL2 regimen. Clin Cancer Res 2016;22:3734–45. 27006492 10.1158/1078-0432.CCR-15-1879

[4] Sarnaik A , Khushalani NI, Chesney JA, Kluger HM, Curti BD, Lewis KD, . Safety and efficacy of cryopreserved autologous tumor infiltrating lymphocyte therapy (LN-144, lifileucel) in advanced metastatic melanoma patients who progressed on multiple prior therapies including anti-PD-1. J Clin Oncol 37: 15s, 2019 (suppl; abstr 2518).

[5] Rosenberg SA , Restifo NP. Adoptive cell transfer as personalized immunotherapy for human cancer. Science 2015;348:62–8. 25838374 10.1126/science.aaa4967 PMC6295668

[6] Stevanović S , Draper LM, Langhan MM, Campbell TE, Kwong ML, Wunderlich JR, . Complete regression of metastatic cervical cancer after treatment with human papillomavirus-targeted tumor-infiltrating T cells. J Clin Oncol 2015;33:1543–50. 25823737 10.1200/JCO.2014.58.9093 PMC4417725

[7] Tran E , Turcotte S, Gros A, Robbins PF, Lu Y-C, Dudley ME, . Cancer immunotherapy based on mutation-specific CD4+ T cells in a patient with epithelial cancer. Science 2014;344:641–5. 24812403 10.1126/science.1251102 PMC6686185

[8] Zacharakis N , Chinnasamy H, Black M, Xu H, Lu Y-C, Zheng Z, . Immune recognition of somatic mutations leading to complete durable regression in metastatic breast cancer. Nat Med 2018;24:724–30. 29867227 10.1038/s41591-018-0040-8 PMC6348479

[9] Tran E , Robbins PF, Rosenberg SA. ‘Final common pathway’ of human cancer immunotherapy: targeting random somatic mutations. Nat Immunol 2017;18:255–62. 28198830 10.1038/ni.3682 PMC6295671

[10] Schumacher TN , Scheper W, Kvistborg P. Cancer Neoantigens. Annu Rev Immunol 2019;37:173–200. 30550719 10.1146/annurev-immunol-042617-053402

[11] Gubin MM , Zhang X, Schuster H, Caron E, Ward JP, Noguchi T, . Checkpoint blockade cancer immunotherapy targets tumour-specific mutant antigens. Nature 2014;515:577–81. 25428507 10.1038/nature13988 PMC4279952

[12] Gros A , Robbins PF, Yao X, Li YF, Turcotte S, Tran E, . PD-1 identifies the patient-specific CD8^+^ tumor-reactive repertoire infiltrating human tumors. J Clin Invest 2014;124:2246–59. 24667641 10.1172/JCI73639 PMC4001555

[13] Pasetto A , Gros A, Robbins PF, Deniger DC, Prickett TD, Matus-Nicodemos R, . Tumor- and neoantigen-reactive T-cell receptors can be identified based on their frequency in fresh tumor. Cancer Immunol Res 2016;4:734–43. 27354337 10.1158/2326-6066.CIR-16-0001 PMC5010958

[14] Duhen T , Duhen R, Montler R, Moses J, Moudgil T, de Miranda NF, . Co-expression of CD39 and CD103 identifies tumor-reactive CD8 T cells in human solid tumors. Nat Commun 2018;9:2724. 30006565 10.1038/s41467-018-05072-0 PMC6045647

[15] Dijkstra KK , Cattaneo CM, Weeber F, Chalabi M, van de Haar J, Fanchi LF, . Generation of tumor-reactive T cells by co-culture of peripheral blood lymphocytes and tumor organoids. Cell 2018;174:1586–98. 30100188 10.1016/j.cell.2018.07.009 PMC6558289

[16] Parkhurst MR , Robbins PF, Tran E, Prickett TD, Gartner JJ, Jia L, . Unique neoantigens arise from somatic mutations in patients with gastrointestinal cancers. Cancer Discov 2019;9:1022–35. 31164343 10.1158/2159-8290.CD-18-1494 PMC7138461

[17] Gattinoni L , Zhong X-S, Palmer DC, Ji Y, Hinrichs CS, Yu Z, . Wnt signaling arrests effector T cell differentiation and generates CD8+ memory stem cells. Nat Med 2009;15:808–13. 19525962 10.1038/nm.1982 PMC2707501

[18] Krishna S , Lowery FJ, Copeland AR, Bahadiroglu E, Mukherjee R, Jia L, . Stem-like CD8 T cells mediate response of adoptive cell immunotherapy against human cancer. Science 2020;370:1328–34. 33303615 10.1126/science.abb9847 PMC8883579

[19] Simoni Y , Becht E, Fehlings M, Loh CY, Koo S-L, Teng KWW, . Bystander CD8+ T cells are abundant and phenotypically distinct in human tumour infiltrates. Nature 2018;557:575–9. 29769722 10.1038/s41586-018-0130-2

[20] Scheper W , Kelderman S, Fanchi LF, Linnemann C, Bendle G, de Rooij MAJ, . Low and variable tumor reactivity of the intratumoral TCR repertoire in human cancers. Nat Med 2019;25:89–94. 30510250 10.1038/s41591-018-0266-5

[21] Vizcardo R , Masuda K, Yamada D, Ikawa T, Shimizu K, Fujii S-I, . Regeneration of human tumor antigen-specific T cells from iPSCs derived from mature CD8(+) T cells. Cell Stem Cell 2013;12:31–6. 23290135 10.1016/j.stem.2012.12.006

[22] Maeda T , Nagano S, Ichise H, Kataoka K, Yamada D, Ogawa S, . Regeneration of CD8αβ T cells from T-cell-derived iPSC imparts potent tumor antigen-specific cytotoxicity. Cancer Res 2016;76:6839–50. 27872100 10.1158/0008-5472.CAN-16-1149

[23] Parkhurst M , Gros A, Pasetto A, Prickett T, Crystal JS, Robbins P, . Isolation of T-cell receptors specifically reactive with mutated tumor-associated antigens from Tumor-infiltrating lymphocytes based on CD137 expression. Clin Cancer Res 2017;23:2491–505. 27827318 10.1158/1078-0432.CCR-16-2680 PMC6453117

[24] Cohen CJ , Zhao Y, Zheng Z, Rosenberg SA, Morgan RA. Enhanced antitumor activity of murine-human hybrid T-cell receptor (TCR) in human lymphocytes is associated with improved pairing and TCR/CD3 stability. Cancer Res 2006;66:8878–86. 16951205 10.1158/0008-5472.CAN-06-1450 PMC2147082

[25] Hong H , Takahashi K, Ichisaka T, Aoi T, Kanagawa O, Nakagawa M, . Suppression of induced pluripotent stem cell generation by the p53-p21 pathway. Nature 2009;460:1132–5. 19668191 10.1038/nature08235 PMC2917235

[26] Li H , Collado M, Villasante A, Strati K, Ortega S, Cañamero M, . The Ink4/Arf locus is a barrier for iPS cell reprogramming. Nature 2009;460:1136–9. 19668188 10.1038/nature08290 PMC3578184

[27] Takahashi K , Yamanaka S. Induction of pluripotent stem cells from mouse embryonic and adult fibroblast cultures by defined factors. Cell 2006;126:663–76. 16904174 10.1016/j.cell.2006.07.024

[28] Shitaoka K , Hamana H, Kishi H, Hayakawa Y, Kobayashi E, Sukegawa K, . Identification of tumoricidal TCRs from tumor-infiltrating lymphocytes by single-cell analysis. Cancer Immunol Res 2018;6:378–88. 29475880 10.1158/2326-6066.CIR-17-0489

[29] Terada K , Kondo K, Ishigaki H, Nagashima A, Satooka H, Nagano S, . Isolation of TCR genes with tumor-killing activity from tumor-infiltrating and circulating lymphocytes in a tumor rejection cynomolgus macaque model. Mol Ther Oncolytics 2021; 24:77–86. 35024435 10.1016/j.omto.2021.12.003 PMC8717465

[30] Nishimura T , Kaneko S, Kawana-Tachikawa A, Tajima Y, Goto H, Zhu D, . Generation of rejuvenated antigen-specific T cells by reprogramming to pluripotency and redifferentiation. Cell Stem Cell 2013;12:114–26. 23290140 10.1016/j.stem.2012.11.002

[31] Kristensen NP , Heeke C, Tvingsholm SA, Borch A, Draghi A, Crowther MD, . Neoantigen-reactive CD8+ T cells affect clinical outcome of adoptive cell therapy with tumor-infiltrating lymphocytes in melanoma. J Clin Invest 2022; 132:e150535. 34813506 10.1172/JCI150535 PMC8759789

[32] Ito T , Kawai Y, Yasui Y, Iriguchi S, Minagawa A, Ishii T, . The therapeutic potential of multiclonal tumoricidal T cells derived from tumor infiltrating lymphocyte-1derived iPS cells. Commun Biol 2021; 4:694. 34099861 10.1038/s42003-021-02195-x PMC8184746

[33] Kim S-M , Bhonsle L, Besgen P, Nickel J, Backes A, Held K, . Analysis of the paired TCR α- and β-chains of single human T cells. PLoS One 2012;7:e37338. 22649519 10.1371/journal.pone.0037338 PMC3359365

[34] Han A , Glanville J, Hansmann L, Davis MM. Corrigendum: linking T-cell receptor sequence to functional phenotype at the single-cell level. Nat Biotechnol 2015;33:210. 10.1038/nbt0215-210c PMC5800857 25658286

[35] Lu Y-C , Zheng Z, Robbins PF, Tran E, Prickett TD, Gartner JJ, . An efficient single-cell RNA-Seq approach to identify neoantigen-specific T cell receptors. Mol Ther 2018;26:379–89. 29174843 10.1016/j.ymthe.2017.10.018 PMC5835023

[36] Yamaguchi S , Hamana H, Shitaoka K, Sukegawa K, Nagata T, Hayee A, . TCR function analysis using a novel system reveals the multiple unconventional tumor-reactive T cells in human breast cancer-infiltrating lymphocytes. Eur J Immunol 2021;51:2306–16. 34171120 10.1002/eji.202049070

[37] Minagawa A , Yoshikawa T, Yasukawa M, Hotta A, Kunitomo M, Iriguchi S, . Enhancing T cell receptor stability in rejuvenated iPSC-derived T cells improves their use in cancer immunotherapy. Cell Stem Cell 2018;23:850–8. 30449714 10.1016/j.stem.2018.10.005

[38] Montel-Hagen A , Seet CS, Li S, Chick B, Zhu Y, Chang P, . Organoid-induced differentiation of conventional T cells from human pluripotent stem cells. Cell Stem Cell 2019;24:376–89. 30661959 10.1016/j.stem.2018.12.011 PMC6687310

[39] Wang Z , McWilliams-Koeppen HP, Reza H, Ostberg JR, Chen W, Wang X, . 3D-organoid culture supports differentiation of human CAR+ iPSCs into highly functional CAR T cells. Cell Stem Cell 2022;29:515–27. 35278370 10.1016/j.stem.2022.02.009 PMC9119152

[40] Vizcardo R , Klemen ND, Islam SMR, Gurusamy D, Tamaoki N, Yamada D, . Generation of tumor antigen-specific iPSC-derived thymic emigrants using a 3D thymic culture system. Cell Rep 2018;22:3175–90. 29562175 10.1016/j.celrep.2018.02.087 PMC5930030

