# Supplementary material for: Reprogramming of Tumor-reactive Tumor-infiltrating Lymphocytes to Human-induced Pluripotent Stem Cells
Source: Cancer Res Commun. 2023 May 25;3(5):917–32. doi: 10.1158/2767-9764.CRC-22-0265 (PMC10211394; doi:10.1158/2767-9764.CRC-22-0265)
Supplement: Table S1 — indicating TIL-iPSCs were established from T cells of low frequency along with major tumor neoantigen specific T cells from patient 3784 [file crc-22-0265-s01.pptx]

## Slide 1
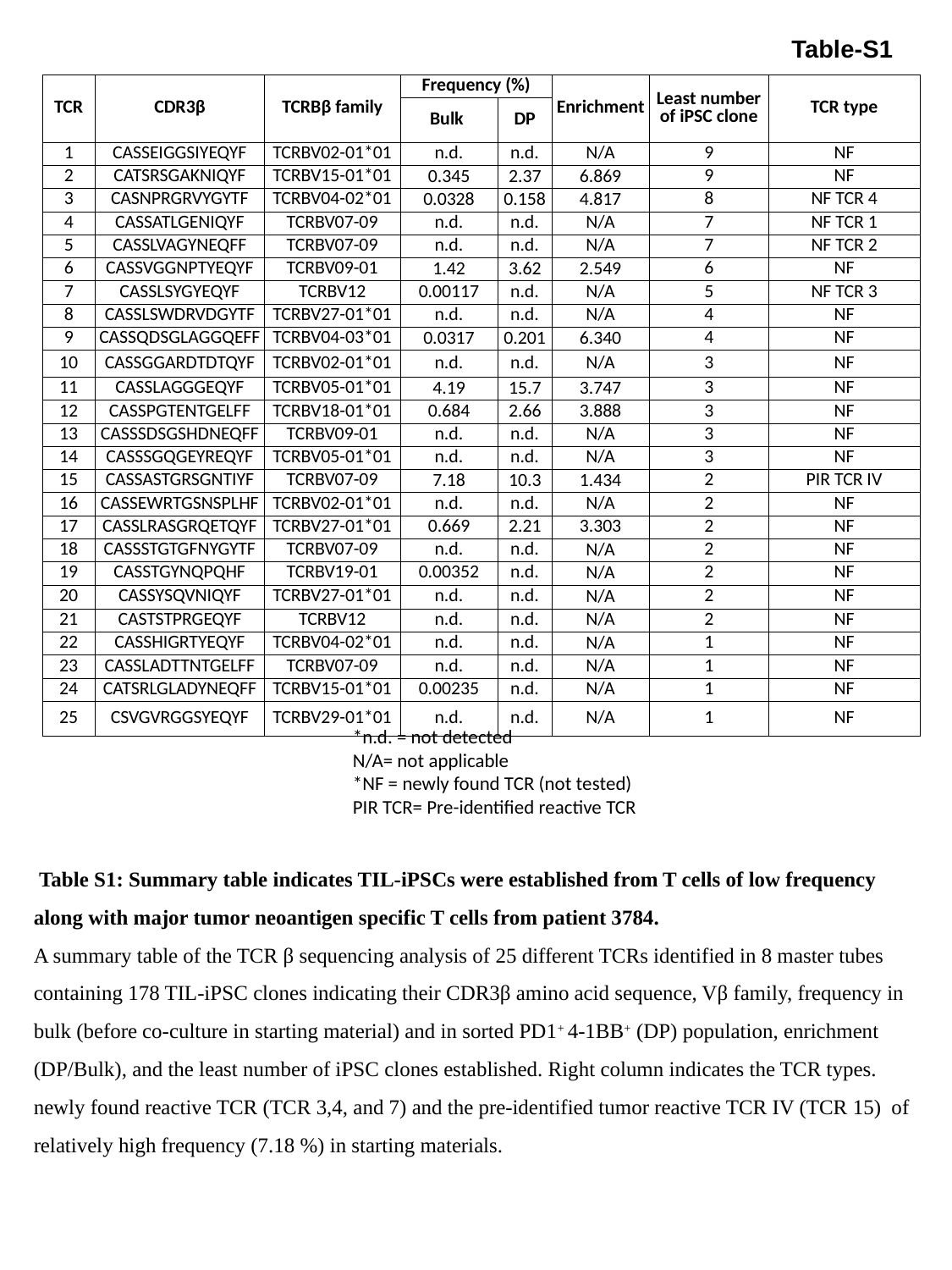

Table-S1
| TCR | CDR3β | TCRBβ family | Frequency (%) | | Enrichment | Least number of iPSC clone | TCR type |
| --- | --- | --- | --- | --- | --- | --- | --- |
| | | | Bulk | DP | | | |
| 1 | CASSEIGGSIYEQYF | TCRBV02-01\*01 | n.d. | n.d. | N/A | 9 | NF |
| 2 | CATSRSGAKNIQYF | TCRBV15-01\*01 | 0.345 | 2.37 | 6.869 | 9 | NF |
| 3 | CASNPRGRVYGYTF | TCRBV04-02\*01 | 0.0328 | 0.158 | 4.817 | 8 | NF TCR 4 |
| 4 | CASSATLGENIQYF | TCRBV07-09 | n.d. | n.d. | N/A | 7 | NF TCR 1 |
| 5 | CASSLVAGYNEQFF | TCRBV07-09 | n.d. | n.d. | N/A | 7 | NF TCR 2 |
| 6 | CASSVGGNPTYEQYF | TCRBV09-01 | 1.42 | 3.62 | 2.549 | 6 | NF |
| 7 | CASSLSYGYEQYF | TCRBV12 | 0.00117 | n.d. | N/A | 5 | NF TCR 3 |
| 8 | CASSLSWDRVDGYTF | TCRBV27-01\*01 | n.d. | n.d. | N/A | 4 | NF |
| 9 | CASSQDSGLAGGQEFF | TCRBV04-03\*01 | 0.0317 | 0.201 | 6.340 | 4 | NF |
| 10 | CASSGGARDTDTQYF | TCRBV02-01\*01 | n.d. | n.d. | N/A | 3 | NF |
| 11 | CASSLAGGGEQYF | TCRBV05-01\*01 | 4.19 | 15.7 | 3.747 | 3 | NF |
| 12 | CASSPGTENTGELFF | TCRBV18-01\*01 | 0.684 | 2.66 | 3.888 | 3 | NF |
| 13 | CASSSDSGSHDNEQFF | TCRBV09-01 | n.d. | n.d. | N/A | 3 | NF |
| 14 | CASSSGQGEYREQYF | TCRBV05-01\*01 | n.d. | n.d. | N/A | 3 | NF |
| 15 | CASSASTGRSGNTIYF | TCRBV07-09 | 7.18 | 10.3 | 1.434 | 2 | PIR TCR IV |
| 16 | CASSEWRTGSNSPLHF | TCRBV02-01\*01 | n.d. | n.d. | N/A | 2 | NF |
| 17 | CASSLRASGRQETQYF | TCRBV27-01\*01 | 0.669 | 2.21 | 3.303 | 2 | NF |
| 18 | CASSSTGTGFNYGYTF | TCRBV07-09 | n.d. | n.d. | N/A | 2 | NF |
| 19 | CASSTGYNQPQHF | TCRBV19-01 | 0.00352 | n.d. | N/A | 2 | NF |
| 20 | CASSYSQVNIQYF | TCRBV27-01\*01 | n.d. | n.d. | N/A | 2 | NF |
| 21 | CASTSTPRGEQYF | TCRBV12 | n.d. | n.d. | N/A | 2 | NF |
| 22 | CASSHIGRTYEQYF | TCRBV04-02\*01 | n.d. | n.d. | N/A | 1 | NF |
| 23 | CASSLADTTNTGELFF | TCRBV07-09 | n.d. | n.d. | N/A | 1 | NF |
| 24 | CATSRLGLADYNEQFF | TCRBV15-01\*01 | 0.00235 | n.d. | N/A | 1 | NF |
| 25 | CSVGVRGGSYEQYF | TCRBV29-01\*01 | n.d. | n.d. | N/A | 1 | NF |
*n.d. = not detected
N/A= not applicable
*NF = newly found TCR (not tested)
PIR TCR= Pre-identified reactive TCR
 Table S1: Summary table indicates TIL-iPSCs were established from T cells of low frequency along with major tumor neoantigen specific T cells from patient 3784.
A summary table of the TCR β sequencing analysis of 25 different TCRs identified in 8 master tubes containing 178 TIL-iPSC clones indicating their CDR3β amino acid sequence, Vβ family, frequency in bulk (before co-culture in starting material) and in sorted PD1+ 4-1BB+ (DP) population, enrichment (DP/Bulk), and the least number of iPSC clones established. Right column indicates the TCR types. newly found reactive TCR (TCR 3,4, and 7) and the pre-identified tumor reactive TCR IV (TCR 15) of relatively high frequency (7.18 %) in starting materials.
