# Supplementary material for: Reprogramming of Tumor-reactive Tumor-infiltrating Lymphocytes to Human-induced Pluripotent Stem Cells
Source: Cancer Res Commun. 2023 May 25;3(5):917–32. doi: 10.1158/2767-9764.CRC-22-0265 (PMC10211394; doi:10.1158/2767-9764.CRC-22-0265)
Supplement: Figure S1 — A summary table of the patient samples used and the schema of the study [file crc-22-0265-s02.pptx]

## Slide 1
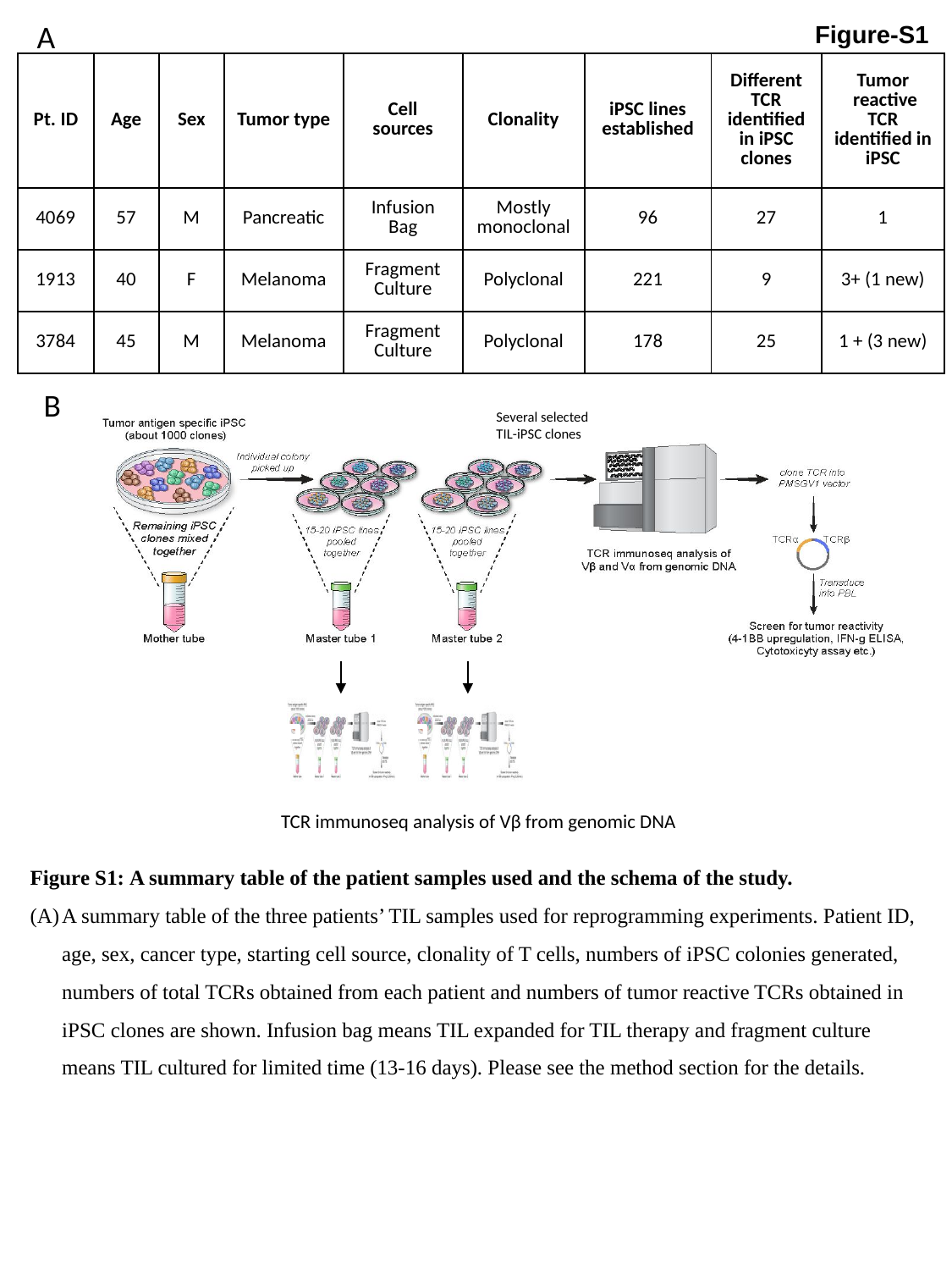

A
Figure-S1
| Pt. ID | Age | Sex | Tumor type | Cell sources | Clonality | iPSC lines established | Different TCR identified in iPSC clones | Tumor reactive TCR identified in iPSC |
| --- | --- | --- | --- | --- | --- | --- | --- | --- |
| 4069 | 57 | M | Pancreatic | Infusion Bag | Mostly monoclonal | 96 | 27 | 1 |
| 1913 | 40 | F | Melanoma | Fragment Culture | Polyclonal | 221 | 9 | 3+ (1 new) |
| 3784 | 45 | M | Melanoma | Fragment Culture | Polyclonal | 178 | 25 | 1 + (3 new) |
B
Several selected TIL-iPSC clones
TCR immunoseq analysis of Vβ from genomic DNA
Figure S1: A summary table of the patient samples used and the schema of the study.
A summary table of the three patients’ TIL samples used for reprogramming experiments. Patient ID, age, sex, cancer type, starting cell source, clonality of T cells, numbers of iPSC colonies generated, numbers of total TCRs obtained from each patient and numbers of tumor reactive TCRs obtained in iPSC clones are shown. Infusion bag means TIL expanded for TIL therapy and fragment culture means TIL cultured for limited time (13-16 days). Please see the method section for the details.

## Slide 2
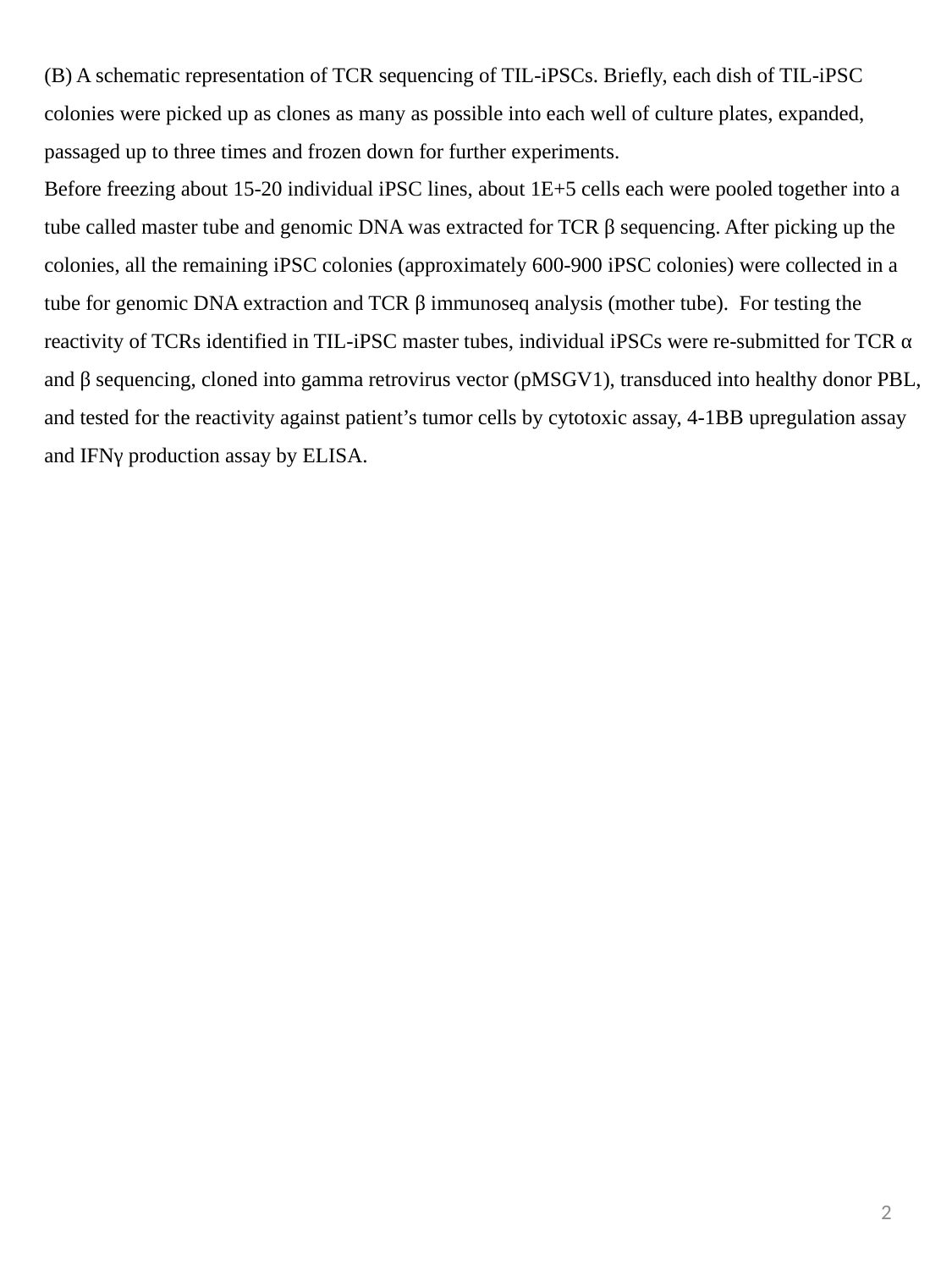

(B) A schematic representation of TCR sequencing of TIL-iPSCs. Briefly, each dish of TIL-iPSC colonies were picked up as clones as many as possible into each well of culture plates, expanded, passaged up to three times and frozen down for further experiments.
Before freezing about 15-20 individual iPSC lines, about 1E+5 cells each were pooled together into a tube called master tube and genomic DNA was extracted for TCR β sequencing. After picking up the colonies, all the remaining iPSC colonies (approximately 600-900 iPSC colonies) were collected in a tube for genomic DNA extraction and TCR β immunoseq analysis (mother tube).  For testing the reactivity of TCRs identified in TIL-iPSC master tubes, individual iPSCs were re-submitted for TCR α and β sequencing, cloned into gamma retrovirus vector (pMSGV1), transduced into healthy donor PBL, and tested for the reactivity against patient’s tumor cells by cytotoxic assay, 4-1BB upregulation assay and IFNγ production assay by ELISA.
2
