# Supplementary material for: Reprogramming of Tumor-reactive Tumor-infiltrating Lymphocytes to Human-induced Pluripotent Stem Cells
Source: Cancer Res Commun. 2023 May 25;3(5):917–32. doi: 10.1158/2767-9764.CRC-22-0265 (PMC10211394; doi:10.1158/2767-9764.CRC-22-0265)
Supplement: Figure S2 — Newly identified TCRs in TIL-iPSCs established from patient 4069 TIL by αCD3/28 Ab stimulation [file crc-22-0265-s03.pptx]

## Slide 1
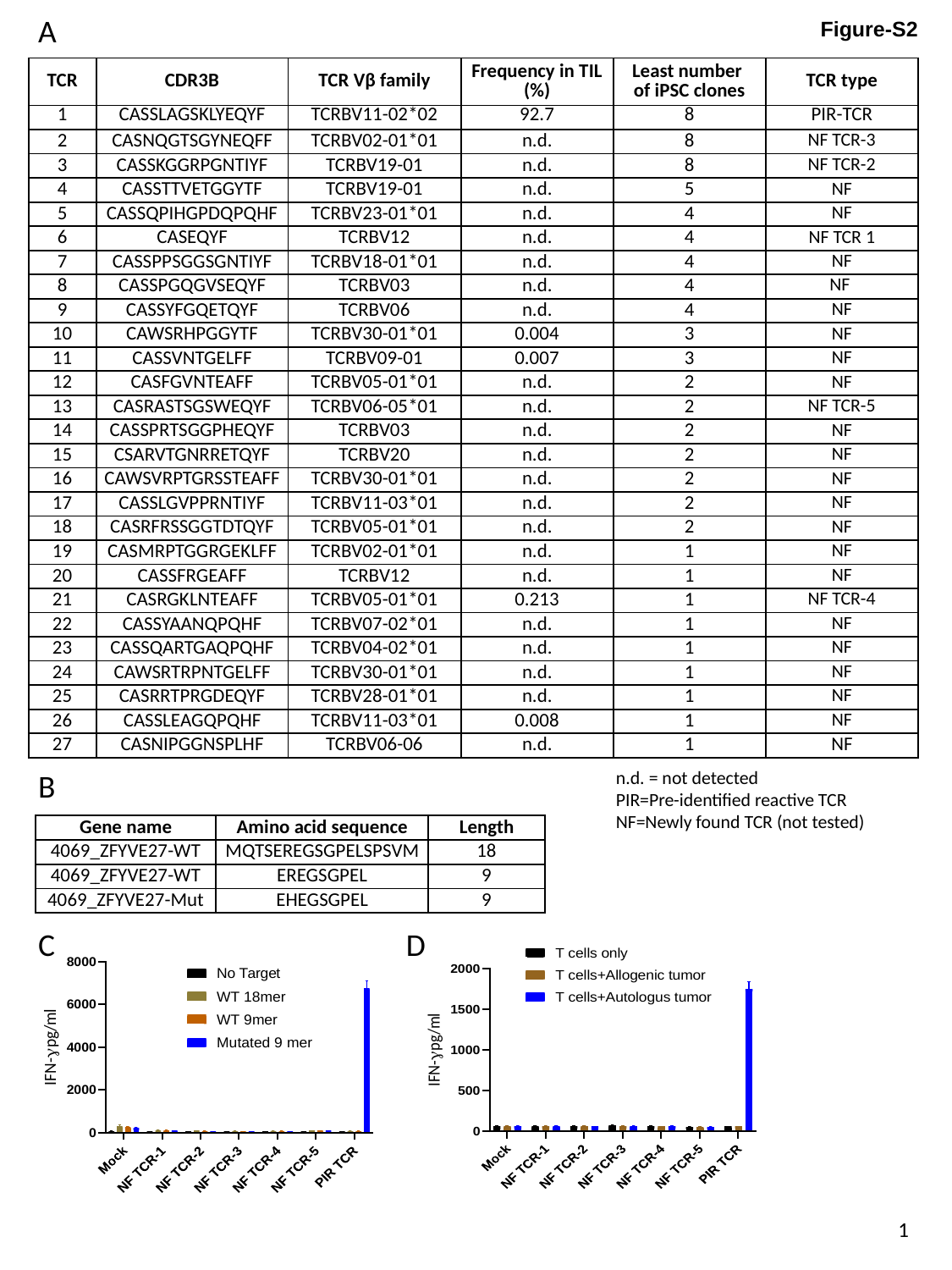

A
Figure-S2
| TCR | CDR3B | TCR Vβ family | Frequency in TIL (%) | Least number of iPSC clones | TCR type |
| --- | --- | --- | --- | --- | --- |
| 1 | CASSLAGSKLYEQYF | TCRBV11-02\*02 | 92.7 | 8 | PIR-TCR |
| 2 | CASNQGTSGYNEQFF | TCRBV02-01\*01 | n.d. | 8 | NF TCR-3 |
| 3 | CASSKGGRPGNTIYF | TCRBV19-01 | n.d. | 8 | NF TCR-2 |
| 4 | CASSTTVETGGYTF | TCRBV19-01 | n.d. | 5 | NF |
| 5 | CASSQPIHGPDQPQHF | TCRBV23-01\*01 | n.d. | 4 | NF |
| 6 | CASEQYF | TCRBV12 | n.d. | 4 | NF TCR 1 |
| 7 | CASSPPSGGSGNTIYF | TCRBV18-01\*01 | n.d. | 4 | NF |
| 8 | CASSPGQGVSEQYF | TCRBV03 | n.d. | 4 | NF |
| 9 | CASSYFGQETQYF | TCRBV06 | n.d. | 4 | NF |
| 10 | CAWSRHPGGYTF | TCRBV30-01\*01 | 0.004 | 3 | NF |
| 11 | CASSVNTGELFF | TCRBV09-01 | 0.007 | 3 | NF |
| 12 | CASFGVNTEAFF | TCRBV05-01\*01 | n.d. | 2 | NF |
| 13 | CASRASTSGSWEQYF | TCRBV06-05\*01 | n.d. | 2 | NF TCR-5 |
| 14 | CASSPRTSGGPHEQYF | TCRBV03 | n.d. | 2 | NF |
| 15 | CSARVTGNRRETQYF | TCRBV20 | n.d. | 2 | NF |
| 16 | CAWSVRPTGRSSTEAFF | TCRBV30-01\*01 | n.d. | 2 | NF |
| 17 | CASSLGVPPRNTIYF | TCRBV11-03\*01 | n.d. | 2 | NF |
| 18 | CASRFRSSGGTDTQYF | TCRBV05-01\*01 | n.d. | 2 | NF |
| 19 | CASMRPTGGRGEKLFF | TCRBV02-01\*01 | n.d. | 1 | NF |
| 20 | CASSFRGEAFF | TCRBV12 | n.d. | 1 | NF |
| 21 | CASRGKLNTEAFF | TCRBV05-01\*01 | 0.213 | 1 | NF TCR-4 |
| 22 | CASSYAANQPQHF | TCRBV07-02\*01 | n.d. | 1 | NF |
| 23 | CASSQARTGAQPQHF | TCRBV04-02\*01 | n.d. | 1 | NF |
| 24 | CAWSRTRPNTGELFF | TCRBV30-01\*01 | n.d. | 1 | NF |
| 25 | CASRRTPRGDEQYF | TCRBV28-01\*01 | n.d. | 1 | NF |
| 26 | CASSLEAGQPQHF | TCRBV11-03\*01 | 0.008 | 1 | NF |
| 27 | CASNIPGGNSPLHF | TCRBV06-06 | n.d. | 1 | NF |
B
n.d. = not detected
PIR=Pre-identified reactive TCR
NF=Newly found TCR (not tested)
| Gene name | Amino acid sequence | Length |
| --- | --- | --- |
| 4069\_ZFYVE27-WT | MQTSEREGSGPELSPSVM | 18 |
| 4069\_ZFYVE27-WT | EREGSGPEL | 9 |
| 4069\_ZFYVE27-Mut | EHEGSGPEL | 9 |
C
D
1

## Slide 2
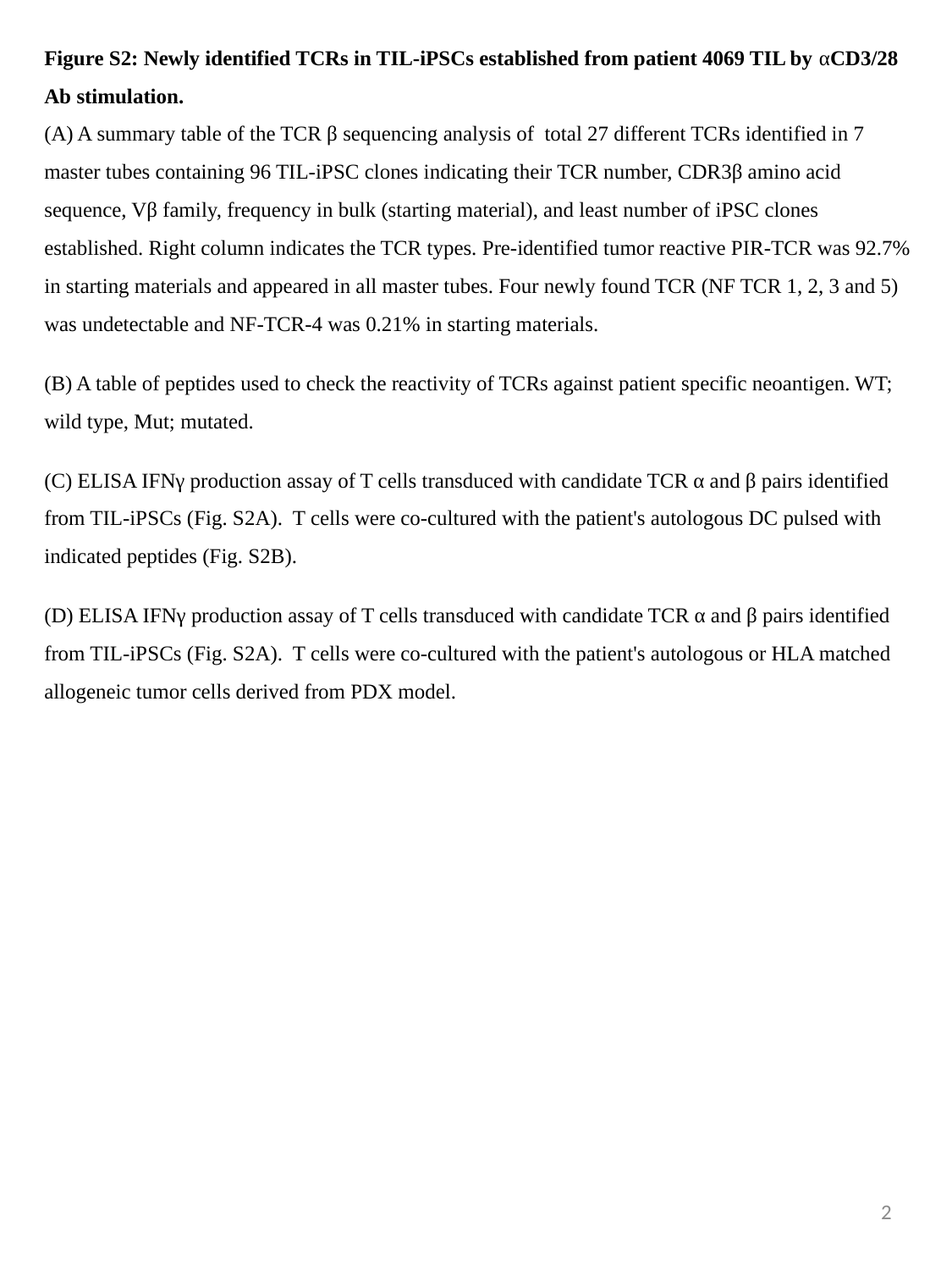

Figure S2: Newly identified TCRs in TIL-iPSCs established from patient 4069 TIL by αCD3/28 Ab stimulation.
(A) A summary table of the TCR β sequencing analysis of total 27 different TCRs identified in 7 master tubes containing 96 TIL-iPSC clones indicating their TCR number, CDR3β amino acid sequence, Vβ family, frequency in bulk (starting material), and least number of iPSC clones established. Right column indicates the TCR types. Pre-identified tumor reactive PIR-TCR was 92.7% in starting materials and appeared in all master tubes. Four newly found TCR (NF TCR 1, 2, 3 and 5) was undetectable and NF-TCR-4 was 0.21% in starting materials.
(B) A table of peptides used to check the reactivity of TCRs against patient specific neoantigen. WT; wild type, Mut; mutated.
(C) ELISA IFNγ production assay of T cells transduced with candidate TCR α and β pairs identified from TIL-iPSCs (Fig. S2A).  T cells were co-cultured with the patient's autologous DC pulsed with indicated peptides (Fig. S2B).
(D) ELISA IFNγ production assay of T cells transduced with candidate TCR α and β pairs identified from TIL-iPSCs (Fig. S2A).  T cells were co-cultured with the patient's autologous or HLA matched allogeneic tumor cells derived from PDX model.
2
