# Supplementary material for: Reprogramming of Tumor-reactive Tumor-infiltrating Lymphocytes to Human-induced Pluripotent Stem Cells
Source: Cancer Res Commun. 2023 May 25;3(5):917–32. doi: 10.1158/2767-9764.CRC-22-0265 (PMC10211394; doi:10.1158/2767-9764.CRC-22-0265)
Supplement: Figure S3 — TIL-Tumor cell co-culture resulted in establishment of TIL-iPSCs from tumor reactive T cell clones from patient 1913 [file crc-22-0265-s04.pptx]

## Slide 1
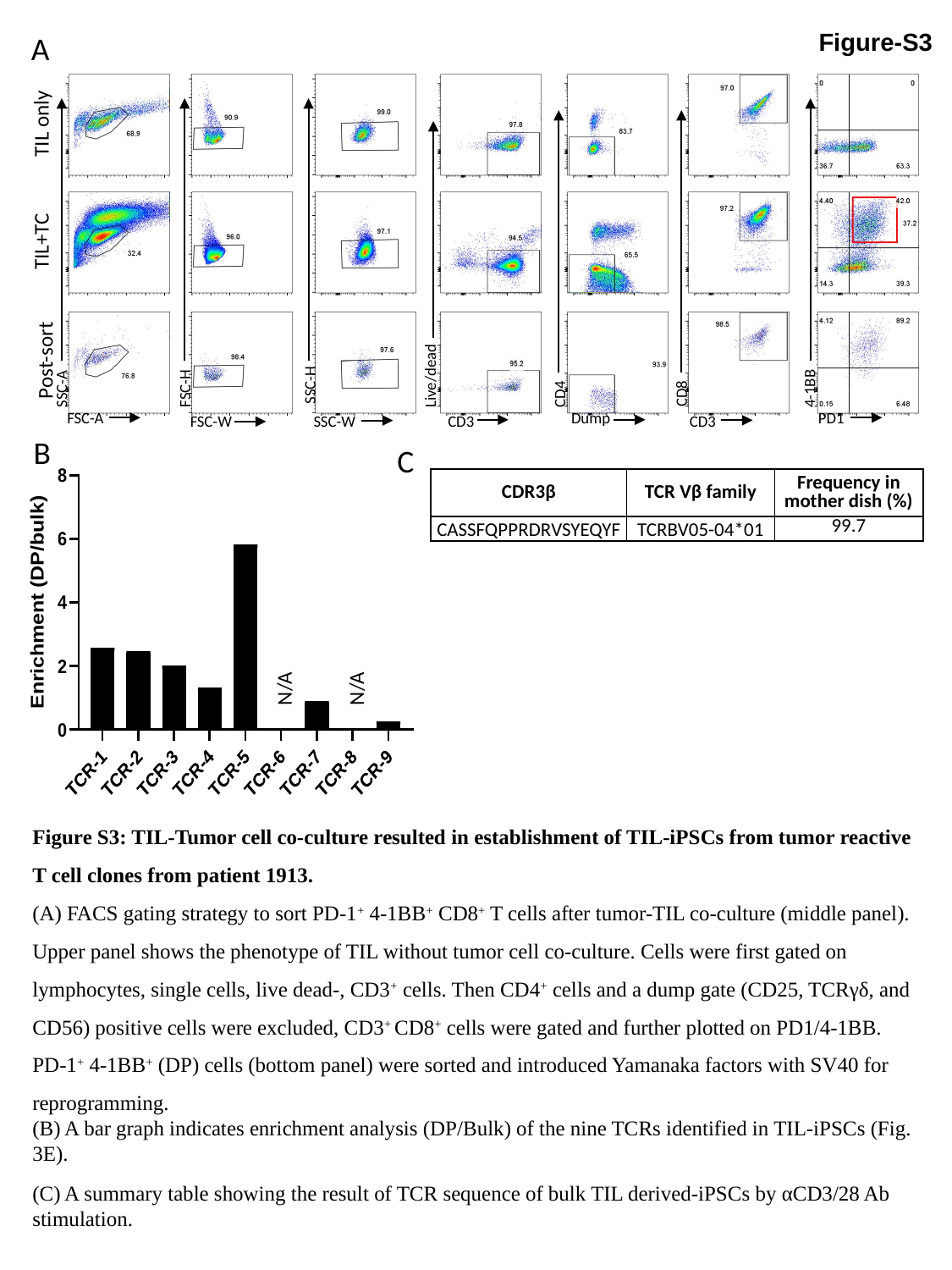

Figure-S3
A
TIL only
TIL+TC
Post-sort
Live/dead
SSC-H
CD8
FSC-H
CD4
SSC-A
4-1BB
FSC-A
Dump
PD1
FSC-W
SSC-W
CD3
CD3
B
C
| CDR3β | TCR Vβ family | Frequency in mother dish (%) |
| --- | --- | --- |
| CASSFQPPRDRVSYEQYF | TCRBV05-04\*01 | 99.7 |
N/A
N/A
Figure S3: TIL-Tumor cell co-culture resulted in establishment of TIL-iPSCs from tumor reactive T cell clones from patient 1913.
(A) FACS gating strategy to sort PD-1+ 4-1BB+ CD8+ T cells after tumor-TIL co-culture (middle panel). Upper panel shows the phenotype of TIL without tumor cell co-culture. Cells were first gated on lymphocytes, single cells, live dead-, CD3+ cells. Then CD4+ cells and a dump gate (CD25, TCRγδ, and CD56) positive cells were excluded, CD3+ CD8+ cells were gated and further plotted on PD1/4-1BB. PD-1+ 4-1BB+ (DP) cells (bottom panel) were sorted and introduced Yamanaka factors with SV40 for reprogramming.
(B) A bar graph indicates enrichment analysis (DP/Bulk) of the nine TCRs identified in TIL-iPSCs (Fig. 3E).
(C) A summary table showing the result of TCR sequence of bulk TIL derived-iPSCs by αCD3/28 Ab stimulation.
