# Supplementary material for: Reprogramming of Tumor-reactive Tumor-infiltrating Lymphocytes to Human-induced Pluripotent Stem Cells
Source: Cancer Res Commun. 2023 May 25;3(5):917–32. doi: 10.1158/2767-9764.CRC-22-0265 (PMC10211394; doi:10.1158/2767-9764.CRC-22-0265)
Supplement: Figure S4 — Characterization of tumor antigen specific TIL-iPSC clone derived from patient 3784 [file crc-22-0265-s05.pptx]

## Slide 1
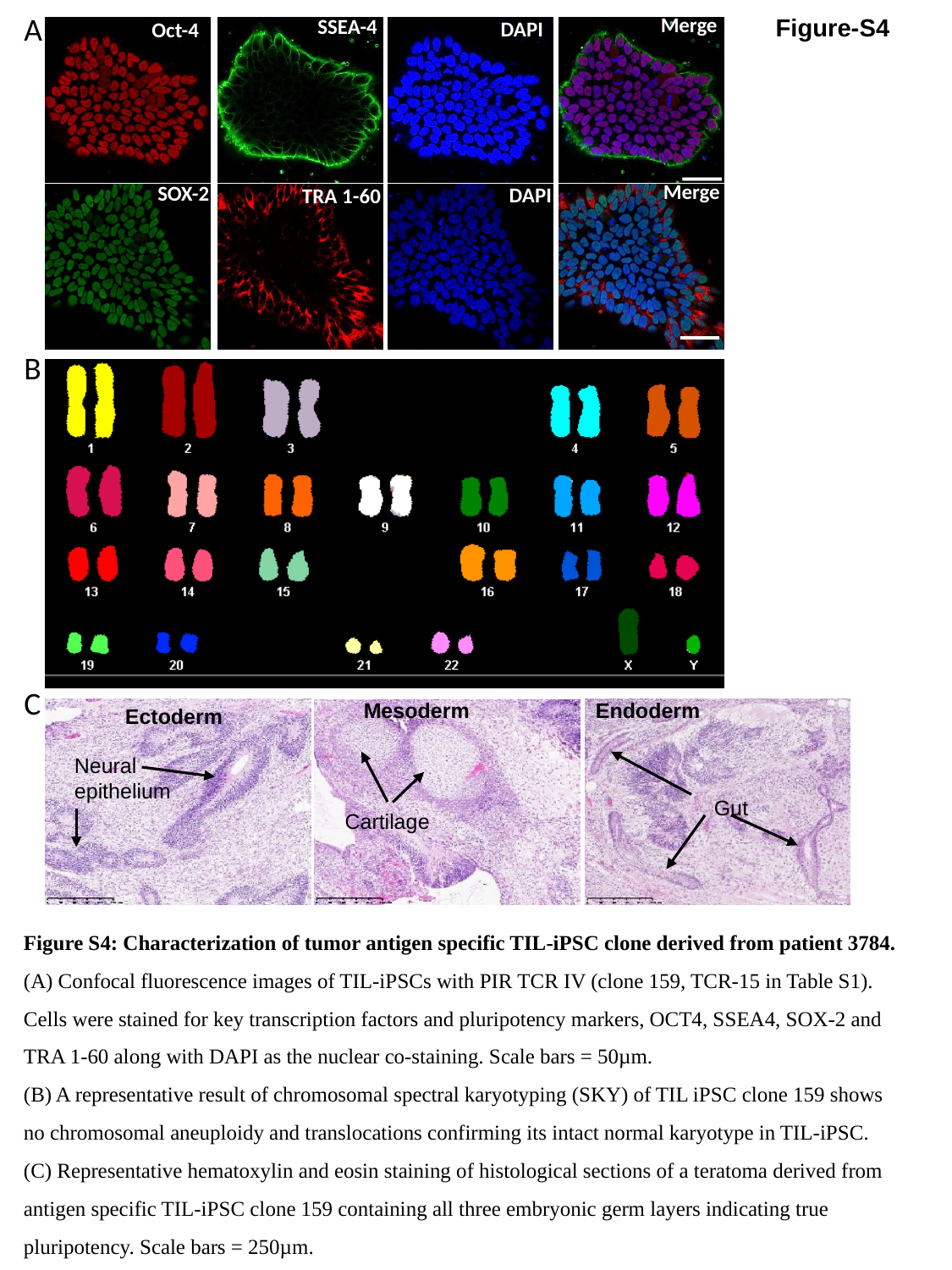

A
Merge
Figure-S4
SSEA-4
DAPI
Oct-4
Merge
SOX-2
DAPI
TRA 1-60
B
C
Endoderm
Mesoderm
Ectoderm
Neural
epithelium
Gut
Cartilage
Figure S4: Characterization of tumor antigen specific TIL-iPSC clone derived from patient 3784.
(A) Confocal fluorescence images of TIL-iPSCs with PIR TCR IV (clone 159, TCR-15 in Table S1). Cells were stained for key transcription factors and pluripotency markers, OCT4, SSEA4, SOX-2 and TRA 1-60 along with DAPI as the nuclear co-staining. Scale bars = 50µm.
(B) A representative result of chromosomal spectral karyotyping (SKY) of TIL iPSC clone 159 shows no chromosomal aneuploidy and translocations confirming its intact normal karyotype in TIL-iPSC.
(C) Representative hematoxylin and eosin staining of histological sections of a teratoma derived from antigen specific TIL-iPSC clone 159 containing all three embryonic germ layers indicating true pluripotency. Scale bars = 250µm.
